# Characterization of particulate depositions collected from archeological monuments in Greece and Cyprus using multiple analytical techniques

**DOI:** 10.1007/s11356-026-37793-x

**Published:** 2026-05-15

**Authors:** Thaleia Gkraikou, Argyri Kozari, Evangelia Vouvoudi, Lambrini Papadopoulou, Vasilios Melfos, Brunella Santarelli, Constantini Samara

**Affiliations:** 1 Department of Chemistry, Laboratory of Environmental Pollution Control, Aristotle University of Thessaloniki, 54124 Thessaloniki, Greece; 2 Department of Chemistry, Laboratory of Polymers and Colours Chemistry and Technology, Aristotle University of Thessaloniki, 54124 Thessaloniki, Greece; 3 Department of Geology, Section of Mineralogy-Petrology-Economic Geology, Aristotle University of Thessaloniki, 54124 Thessaloniki, Greece; 4 The Cyprus Institute, Science and Technology in Archaeology and Culture Research Center (STARC), Konstantinou Kavafi 20, Aglantzia, 2121 Nicosia, Cyprus

**Keywords:** Cultural heritage, Geochemical composition, Airborne dust, Dry deposition, Thessaloniki, Nicosia

## Abstract

Morphological and geochemical characterization of the particulate depositions collected from the surfaces of urban monuments located in two Mediterranean cities characterized by high levels of atmospheric particulate matter (PM), namely Thessaloniki in Greece and Nicosia in Cyprus, was carried out using multiple analytical techniques. The geochemical composition of bulk samples was found to be dominated by CaO and SiO_2_ with lower proportions of Al_2_O_3_, Fe_2_O_3_, and MgO suggesting influence from local and/or transported dust (road dust, Saharan dust). The effect of traffic was evident on the sides of monuments oriented towards busy roads with higher concentrations of anthropogenic elements such as Zn, Ba, Cu, V, and Ni. Gypsum was detected in a few samples only and is possibly attributed to sulfation process and/or windblown Saharan dust. Notably, a wide spectrum of low- and high-molecular-weight organic compounds was also detected (aliphatic hydrocarbons and organic compounds containing carbonyl- and carboxyl-groups, urea and nitrogen-containing biomass, amino acids, fatty acids and lipids, plant residues, synthetic polymer residues, and combustion products) suggesting the presence of various natural and anthropogenic sources. The results obtained in the study may serve as a guide for the development of appropriate strategies for protection and conservation of the urban architectural heritage in the two countries.

**Graphical Abstract:**

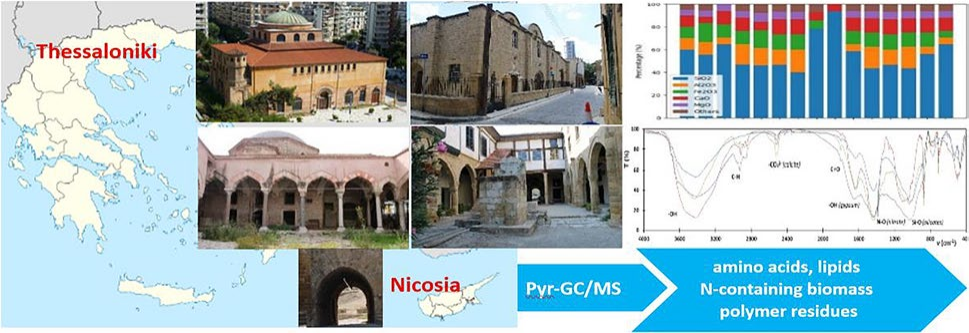

**Supplementary Information:**

The online version contains supplementary material available at 10.1007/s11356-026-37793-x.

## Introduction

Atmospheric pollution plays a key role in the processes of deterioration of monuments and buildings of historical and artistic value, particularly in city centers where traffic is the major source of pollution (Brimblecombe and Grossi 2005; Sabbioni 1995). Atmospheric pollutants like sulfur dioxide (SO_2_), ground-level ozone (O_3_), nitric acid (HNO_3_), acid rain, and airborne particulate matter (PM), defined as all suspended particles that can be found in the atmosphere, may induce significant damage to monuments by causing corrosion, surface degradation, and structural deterioration (https://unece.org/media/news/378562). The measures and policies for atmospheric pollution reduction in Europe, the richest region in terms of sites of Cultural Heritage protected by UNESCO (UNESCO, World Heritage Statistics http://whc.unesco.org/en/list/stat), have cut off the SO_2_ levels, and consequently, its impact on materials is drastically reduced. Indeed, in 1980, the number of European UNESCO sites in danger was extremely high (94% for limestone, 54% for copper, and 1% for bronze) while in 2010, these sites did not exceed the tolerable value of surface recession and corrosion. However, PM is highlighted as the main factor responsible for material corrosion while future scenarios show that recession and mass loss in the case of limestone, bronze, and copper in the Balkan area will exceed the acceptable limits (Di Turo et al. 2016). Fine particles such as PM_2.5_ can remain airborne for longer and penetrate deeper into the pores of the stone. These fine particles are more likely to be carried by air currents into sheltered areas where they can accumulate. The high surface-area-to-volume ratio of fine particles also makes them more chemically reactive. Larger particles (e.g., PM_10_) tend to settle more quickly due to gravity and are often found on horizontal surfaces. Both sizes contribute to the formation of damaging crusts and the overall aesthetic degradation of monuments. Due to higher atmospheric PM levels, urban environments are potentially more harmful to vulnerable archeological heritage even if covered.

Dry deposition of airborne PM onto historic surfaces usually implies both visual changes and chemical weathering (Cabello-Briones et al. 2020). Deposited PM, that is rich in metals and metal oxides, acts as catalyst in the sulfation of limestone, i.e., the transformation of calcium carbonate (calcite) into calcium sulfate dihydrate (gypsum), which, together with embedded carbonaceous particles, consequently, forms the black crusts on the stone surface (Fermo et al. 2015). The resulting salts, usually less soluble, are likely to generate high crystallization pressures leading to detachment of the formed black crusts together with the stone from the surface of the monuments (Moropoulou et al. 1998; Cultrone et al. 2004 & 2008; McAlister et al. 2008; Ruffolo et al. 2015; La Russa et al. 2017; Mitsos et al. 2022).

Research on the composition of particulate depositions provides valuable insight into the major causes of pollution, permitting an identification of the possible pollution sources in the area around a monument. Particulate depositions on monuments, either alone, or along with damaged layers and/or the underlying stone, have been studied across numerous European heritage sites, including major cathedrals and historic structures in Norway (Nava et al. 2010; Ozga et al. 2014), Italy (Fermo et al. 2015 & 2018), Hungary (McAlister et al. 2006 & 2008), Spain (Cabello-Briones et al. 2020), Poland (Marszałek et al. 2024), and Greece (Samara et al. 2020).

The aim of the present study was an exhaustive characterization of particulate depositions from archeological monuments located in two Mediterranean cities that are experiencing high airborne PM levels, Thessaloniki in Greece and Nicosia in Cyprus. To the best of our knowledge, this is the first time that particulate depositions from the surface of urban monuments in the two countries are characterized by employing such a multi-analytical approach.

## Materials and methods

### Description of sites and monuments

Thessaloniki (40° 38′ 11.99″ N, 22° 56′ 31.79″ E), the second largest city in Greece and one of the largest urban agglomerations in the Balkans, has been historically faced with severe air pollution from airborne PM originating mainly from road dust and combustion sources such as vehicular traffic and biomass burning (Argyropoulos et al. 2017). The frequency of Saharan dust episodes in Thessaloniki is lower than in other cities located in southern Greece (Karagkiozidis et al. 2017). Nicosia (35°10′0.01″N, 33°22′0.01″E), the capital and largest city of Cyprus, in the Eastern Mediterranean basin, also experiences high airborne PM levels deriving from long-range transport of Saharan dust frequently occurring in spring and autumn (Achilleos et al. 2014), as well as from traffic, domestic heating, mineral dust, sea salt, and heavy oil combustion (Bimenyimana et al. 2023). Data concerning the deposition rates of atmospheric dust at the two cities are scarce and incomparable. The annual atmospheric dust deposition flux in the Cretan Sea, in the Eastern Mediterranean, was estimated at about 13 g/m^2^ (Theodosi et al. 2019). Correspondingly, the annual total dry deposition flux of gaseous and particulate phases of chloride (HCl + p-Cl), oxidized nitrogen (NO + NO_2_ + HNO_3_ + p-NO_3_), reduced nitrogen (NH_3_ + p-NH_4_), and sulfur (SO_2_ + p-SO_4_) in Thessaloniki exceeded 3 g/m^2^ with 70–90% of dry deposition being driven by gases (Anatolaki & Tsitouridou 2007).

A map showing the sampling sites is provided in Supplementary Material.

Two monuments were sampled in Thessaloniki, Greece: (a) the early Christian Church of Hagia Sophia, a monument listed as a UNESCO World Heritage Site, and (b) the Mosque of Hamza Bey, a fifteenth-century Ottoman Mosque. Additionally, three monuments were sampled in Nicosia, Cyprus: (a) the Hadjigeorgakis Kornesios House, an important surviving urban architecture building from the end of the eighteenth century, (b) the Greek Orthodox Church of Archangel Michael Trypiotis, and (c) the Paphos Gate, one of the three entrances to the Venetian walls surrounding Nicosia. More information about the monuments is provided in Supplementary Material.

### Sample collection

A summary description of the samples collected is provided in Table 1.

**Table 1:** Description of samples of particulate deposits collected from monuments in Thessaloniki, Greece, and Nicosia, Cyprus

Sample code	Monument	City	Orientation of sampled surface	Height a.g.l. (m)	Substrate material	Sheltered point	Previous restoration
DS1	Hagia Sophia Church	Thessaloniki	West	0.3	Limestone	No	No
DS2	Hagia Sophia Church	Thessaloniki	West	0.2	Marble	Partially	Yes
DS3	Hagia Sophia Church	Thessaloniki	South	1	Ceramic bricks	Partially	Yes
DS4	Hagia Sophia Church	Thessaloniki	South	2	Stone	Partially	Yes
DB1	Hamza Bey Mosque	Thessaloniki	West	0.2	Marble	Partially	Yes (> 10 y)
DB2	Hamza Bey Mosque	Thessaloniki	Southwest	0.2	Limestone	Partially	Yes (> 10 y)
DB4	Hamza Bey Mosque	Thessaloniki	South	3	Metallic casing	Partially	Yes (> 10 y)
DB5	Hamza Bey Mosque	Thessaloniki	West	1	Ceramic stones	Yes	No
DB6	Hamza Bey Mosque	Thessaloniki	West	0.2	Limestone	Partially	No
DB7	Hamza Bey Mosque	Thessaloniki	West	0.2	Limestone	Partially	No
DB8	Hamza Bey Mosque	Thessaloniki	Northwest	0.7	Limestone	Partially	No
DM2a	Archangel Michael Trypiotis Church	Nicosia	South	0.4	Stone	Partially	-
DM3a	Archangel Michael Trypiotis Church	Nicosia	West	1	Stone	No	-
DM3b	Archangel Michael Trypiotis Church	Nicosia	West	2	Marble	Partially	-
DK4	Hadjigeorgakis Kornesios House	Nicosia	West	3	Limestone	Partially	-
DK5	Hadjigeorgakis Kornesios House	Nicosia	Northwest	3	Limestone	Partially	-
DK8	Hadjigeorgakis Kornesios House	Nicosia	Northeast	3	Limestone	Partially	-
DK11	Hadjigeorgakis Kornesios House	Nicosia	East	3	Limestone	Yes	-
DK12	Hadjigeorgakis Kornesios House	Nicosia	Atrium	1	Marble	No	-
DK13	Hadjigeorgakis Kornesios House	Nicosia	East Atrium	0.5	Marble	No	-
DP0	Paphos Gate	Nicosia	Southwest	0	Limestone/Local calcite	Yes	-

**Fig. 1:**
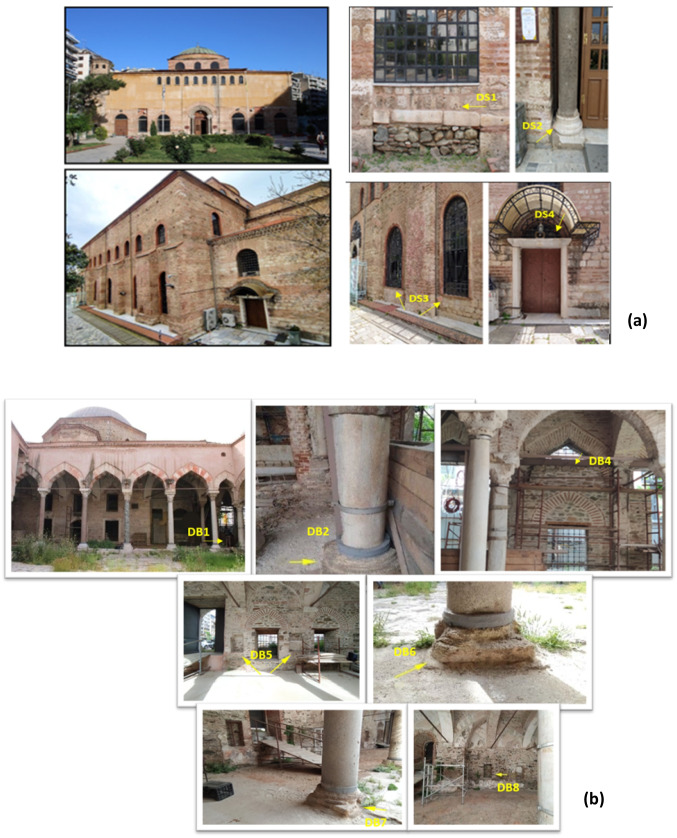
The sampled monuments in Thessaloniki, Greece, with sampling points: **a** the Church of Hagia Sophia, **b** the Hamza Bey Mosque (sample codes as in Table 1)

**Fig. 2:**
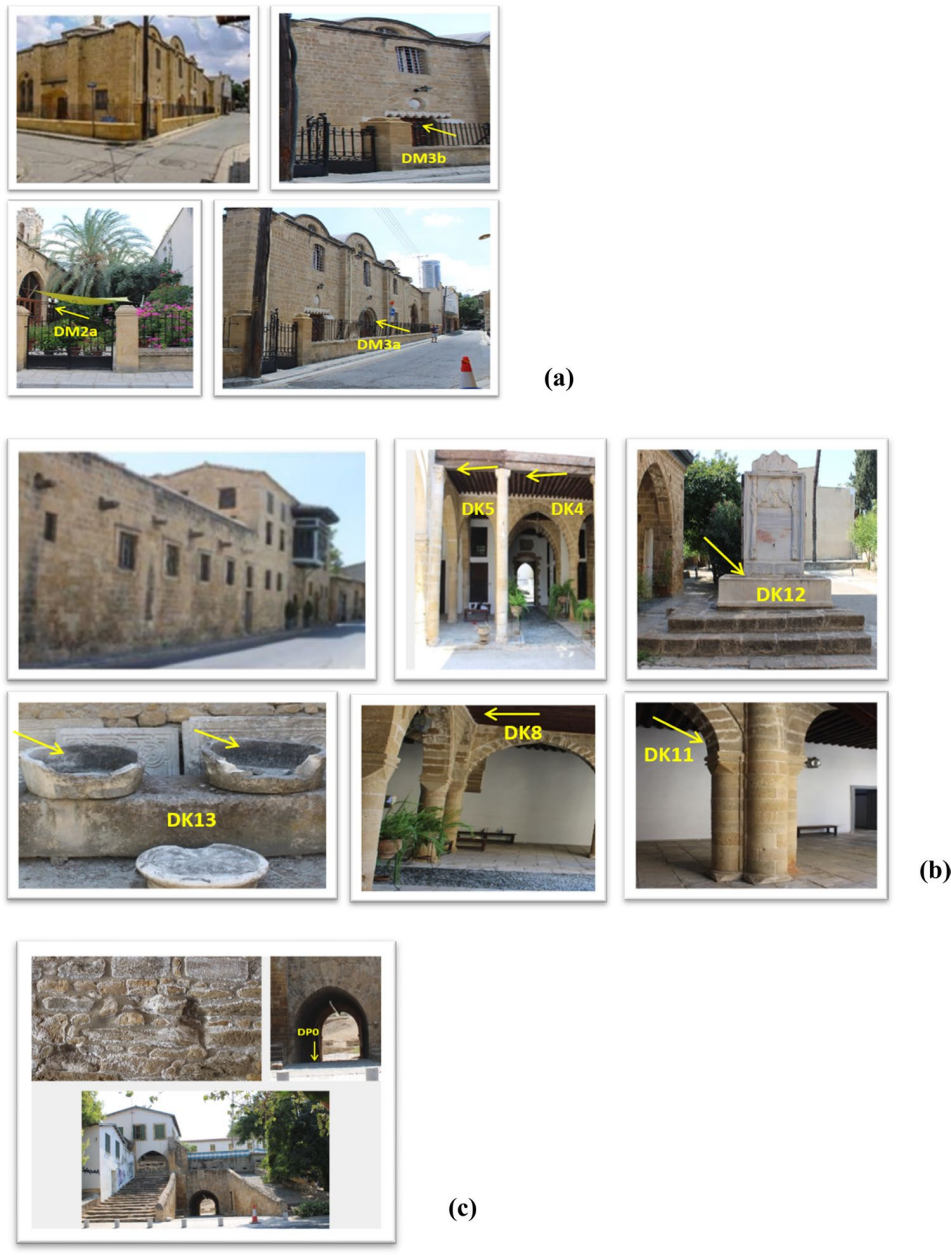
The sampled monuments in Nicosia, Cyprus, with sampling points: **a** the Archangel Michael Trypiotis Church, **b** the House of Hadjigeorgakis Kornesios, **c** the Paphos Gate (sample codes as in Table 1)

Sampling of particulate deposits from the Thessaloniki monuments took place in May 2020. Four samples were collected from the Church of Hagia Sophia (Fig. 1a) and 7 samples from the Hamza Bey Mosque (Fig. 1b). Sampling from the Nicosia monuments took place in August 2020. Three samples were collected from the Church of Michael Trypiotis (Fig. 2a). Six samples were collected from the Hadjigeorgakis Kornesios House (Fig. 2b). Lastly, only one sample could be taken from Paphos Gate. The sample was collected from the ground under the Gate (Fig. 2c); therefore, it was assumed be consisting of soil dust and possibly deteriorated construction material, i.e., friable porous stone (mainly limestone and sandstone) that during summer loses its moisture, forming a multitude of cracks.

In most cases, samples were collected from partially sheltered surfaces. Surfaces on all sides of each monument were selected, as far as possible. The altitude of sampling points ranged between 0.2 and 3 m above ground level. Samples, containing the dry particulate deposition and possibly damaged substrate and mortar material, were collected using a soft brush according to Samara et al. 2020. Collected samples were placed in plastic bags (LDΡΕ) and transported to the laboratory, where they were air-dried for 24 h, homogenized, and weighed using a five-digit analytical balance (KERN 870). Finally, the homogenized samples were subjected to dry sieving to separate the size fraction ≤ 100 μm for subsequent characterization and analysis.

### Methods of sample characterization and analysis

A multi-analytical diagnostic approach was employed for sample characterization and analysis integrating different and complementary techniques. Electron scanning microscopy coupled with energy dispersion spectroscopy (SEM-EDS) was used for sample imaging and elemental microanalysis of individual particles. X-ray fluorescence spectroscopy (XRF) was employed to determine the bulk elemental composition of samples. Ion chromatography (IC) was employed to determine the water-soluble concentrations of inorganic and organic anions (chlorides, sulfates, nitrates, acetates, formates, and oxalates) and cations (sodium, potassium, calcium, magnesium). Fourier transform IR spectroscopy (FT-IR) was used to identify organic and inorganic materials by detecting characteristic functional groups. Finally, pyrolysis–gas chromatography/mass spectrometry (Py-GC/MS) was employed to characterize complex, insoluble, or high-molecular-weight organic materials. Detailed information about the methods used is provided in Supplementary Material.

## Results and discussion

### Morphological, mineralogical, and geochemical characteristics

In all samples, SEM imaging revealed the occurrence of particles of mostly angular to subangular shape, sometimes exhibiting elongate morphologies, with sizes ranging from a few μm up to over 100 μm (Figs. S1 a and S1b).

**Fig. 3:**
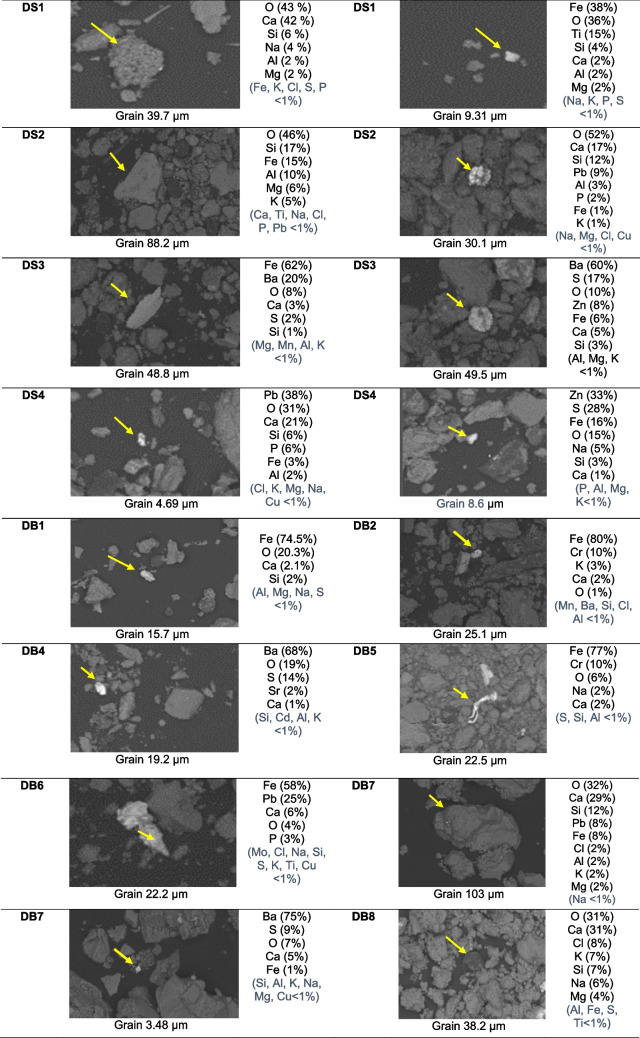

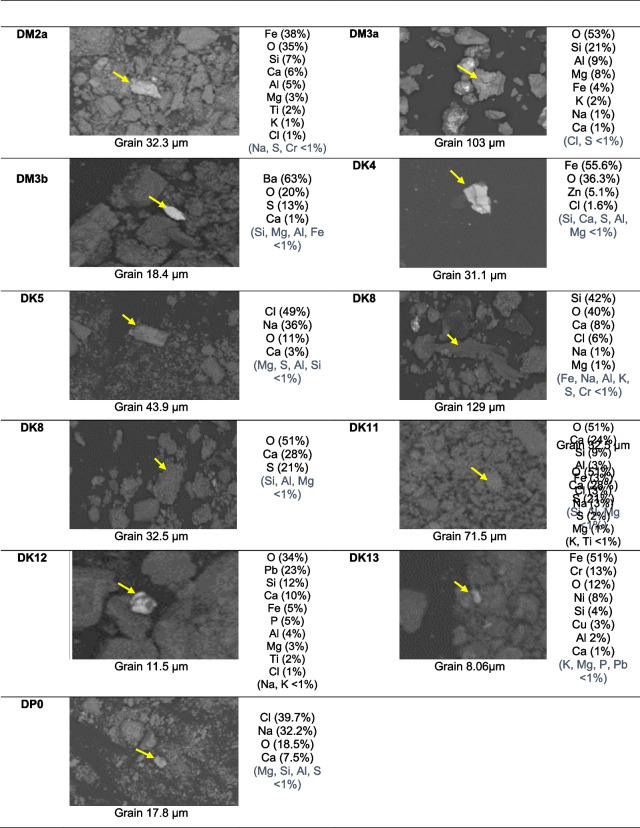
**a** Semiquantitative composition obtained by SEM-EDS for single particles in deposition samples from the Church of Hagia Sophia (DS1–DS4) and the Mosque of Hamza Bey (DB1–DB8) in Thessaloniki, Greece (sample codes as in Table 1). **b** Semiquantitative composition obtained by SEM-EDS for single particles in deposition samples from the Hadjigeorgakis Kornesios House (DK4–DK13), the Church of Archangel Michael Trypiotis (DM2a–DM3b), and the Paphos Gate (DP0) in Nicosia, Cyprus (sample codes as in Table 1)

The semiquantitative composition of single grains, acquired using SEM-EDS, is shown in Fig. 3 a and b, for the monuments of the two cities, respectively.

In the *Church of Hagia Sophia*, samples DS1 and DS2 from limestone and marble substrates at the west side of the monument exhibited similar geochemical composition dominated by aluminosilicate phases with high Ca and Fe content, suggesting inputs from urban dust. Interestingly, sample DS4, from the restored stone, indicated small-sized Pb-rich and Zn-rich particles. These metals are related to vehicular exhaust emissions and/or resuspension of road dust containing elements from brake abrasion, automotive catalysts, and mechanical wear (Belfiore et al. 2013; Ozga et al. 2014; Sýkorová et al. 2017). It is worth noting that metals of anthropogenic origin such as Cr, Zn, and Cu can act as catalysts in the black crust formation process (Fermo et al. 2015). Most samples from the *Hamza Bey Mosque* (DB1, DB2, DB5, and DB6 from marble, limestone, and ceramic stone surfaces) were characterized by Fe-rich particles (52–80%). In addition, DB2, DB5, and DB6 exhibited noticeable Cr and Pb contents. Sample DB7, also from a limestone surface, was characterized by Si-rich and Ca-rich particles with lower contents of Pb, Fe, K, and Mg, suggesting inputs from airborne dust originating from crustal and anthropogenic sources. This geochemical composition of particulate deposits is rather similar to the mineralogical composition of airborne PM and road dust in Thessaloniki that both were found to be dominated by calcite and quartz with lower amounts of gypsum and/or anhydrite in PM10, albite and dolomite in road dust (Samara et al. 2020 and references herein). Furthermore, Fe oxides (γ-Fe_2_O_3_) and other metal-rich particles were found in airborne PM from central Thessaloniki, highlighting the impact from vehicular emissions and heavy oil combustion, as well as tire and brake wear (Grigoratos et al. 2014). However, the minerals detected in the particulate deposits from the monuments may also be partially attributed to the decay of the building materials.

In *Michael Trypiotis Church*, particles rich in Si, Al, Mg, and Fe were prevalent in sample DM3a suggesting potential presence of Saharan dust particles which are dominated by quartz, calcite, dolomite, and clay minerals (Jeong 2024). In *Hadjigeorgakis Kornesios House*, Fe-rich particles were prevalent in sample DK4, while Zn was also detected on a medium-sized grain suggesting potential presence of tire wear particles. Sample DK8 showed high content in quartz, the major component of Sahara dust, while smaller particles, rich in Ca and S, were also detected suggesting potential occurrence of gypsum grains, also present in Saharan dust (Jeong 2024), or formed through sulfation of limestone. Sample DK13, from a low-height surface, was characterized by high contents of Fe–Cr oxides with lower contents of Ni and Cu suggesting inputs from airborne dust originating from traffic-related and other combustion sources.

The presence of Ba-rich particles along with appreciable contents of S, implying the occurrence of barite (BaSO_4_), was apparent in samples from the Hagia Sophia Church, the Hamza Bey Mosque, and the Michael Trypiotis Church including DS3 and DB4, collected from previously restored surfaces, as well as DB7 and DM3b, collected from rather non-restored surfaces (Fig. S1). BaSO_4_ can result from the use of barium hydroxide (Ba(OH)_2_) in conservation interventions. It is well known that Ba(OH)_2_ is one of the most widely used consolidation agents in the nineteenth and twentieth centuries for porous limestones (Doehne & Price 2010), and its use on several monuments in Europe, including the marbles of the Galerius Arch in Thessaloniki, has been documented (Tesser et al. 2022). Other potential sources of Ba could be tire friction on the road, as suggested for historic buildings most exposed to traffic (Belfiore, et al. 2013; Ozga et al. 2014; Sýkorová et al. 2017; Pozo-Antonio et al. 2022), whereas potential emissions from the inefficient burning of Ba-rich coals (Marszałek et al. 2024) should be excluded since coal burning is not applied in either Thessaloniki or Nicosia.

Although Thessaloniki is a coastal city, Na and Cl, indicative of the presence of salts of marine origin (halite), were detected only in sample DB8 from the Hamza Bey Mosque. On the contrary, Na- and Cl-rich particles were found in Hadjigeorgakis Kornesios House in Nicosia, particularly in DK5, as well as in samples DK8, DK11, and DP0, despite the relatively long distance of Nicosia from the sea (17 km). This agrees with other studies; for instance, sea-salt aerosols were widely found embedded in the black crusts on carbonate stones in Granada, Spain, ca. 50 km from the Mediterranean coast (Pozo-Antonio et al. 2022).

### Elemental composition of bulk samples

The geochemical composition of selected particulate deposition samples obtained by XRF analysis of fused samples is shown in Fig. S2.

The most abundant element oxides were found to be CaO and SiO_2_ with lower proportions of Al_2_O_3_, Fe_2_O_3_, and MgO. The highest abundance of SiO_2_ (39–47%) was observed in samples DS1, DS3, and DS4 from the Hagia Sophia Church in Thessaloniki, while the highest abundance of CaO was found in samples DB6 and DB7 from the Hamza Bey Mosque in Thessaloniki (50 and 53%, respectively), as well as in samples DK5 from the Hadjigeorgakis Kornesios House and DP0 from the Paphos Gate in Nicosia (41% and 36%, respectively). SiO_2_ and CaO in airborne PM are considered dust indicators since high concentrations of SiO_2_ are commonly associated with desert-origin dust, while CaO indicates the presence of soil-derived particles. Nevertheless, the high abundance of CaO in deposition samples may also suggest the presence of calcium carbonate originating from substrate material, as most of the samples mentioned above were collected from limestone surfaces.

The elemental composition of the particulate deposition samples, analyzed with the p-XRF, is shown in Fig. S3.

In agreement with the XRF analysis of fused samples, the prevalent elements in samples DS1, DS2, DS3, and DS4 followed the order Ca > Fe > Si, while a different order was observed for DB7 (Ca > Mg > Si) and DB8 (Ca > Fe > K). In most samples, Ca, Fe, and Si were followed by K. Notably, Ca, Fe, Si, Al, and Ti were found at higher concentrations in samples collected from low-height points. Trace elements varied among the different sides of the monuments and the heights of sampling points. Sample DS4, from a point 2 m a.g.l. on the south side of the Hagia Sofia Church, exhibited the highest concentrations of metal(loid)s such as As, Sb, Zn, Pb, Ni, Mn, V, and Cr. Sample DB1, taken from the low-height marble bases of the facade columns of the Hamza Bey Mosque, at the west side of the monument in a spot restored more than 10 years ago, exhibited the highest concentration of Pb (2.1%). Notably, sample DB5, taken from the ceramic stones at 1 m a.g.l. on the west side of the Hamza Bey Mosque, exhibited the highest S content among all samples from Thessaloniki’s monuments (4.8%).

In most samples from Nicosia monuments (DM2a, DM3a, DM3b, DK4, DK5, DK11, DK12, DK13) the prevalent elements followed the order Ca > Fe > Si. In most samples, Ca, Fe, and Si were followed by S, which exceeded Si in DK8, on the northeastern side. The highest S content was found in samples DM3b and DK8 (4.9% and 4.7%, respectively). The only sample that exhibited noticeable Cl content (2.3%), together with high values of Ca and other crustal elements (Fe, Si), was DP0 collected from the ground under the Paphos Gate.

It is worth noting that phosphorus (P) was detected in almost all deposition samples, primarily in those from Thessaloniki’s monuments (0.34% in DS2 and 0.31% in DS1) and secondarily in those from Nicosia’s monuments (0.25% in DK13 and 0.19% in DM3a). The presence of P in particulate depositions on monument surfaces could be related to bird droppings (Samara et al. 2020). Bird excreta can cause chemical deterioration of masonry surfaces through the introduction of acids (Spennemann et al. 2017). However, other P sources such as combustion emissions, mineral dust, primary biological aerosol particles, and sea salt should not be excluded. Bioaerosols and dust were suggested to be the dominant sources of organic P in atmospheric particles in Eastern Mediterranean (Violaki et al. 2021).

Correlation coefficients between elemental species determined in particulate depositions from monuments of Thessaloniki and Nicosia are provided in Tables S1 and S2.

High correlation coefficients were found between crustal elements, such as Al-Si (0.95), Mn-Fe (0.93), Ti-Fe (0.92), Si-Fe (0.90), Al-Fe (0.89), Al-Ti (0.86), Ti-V (0.79), Si-P (0.64), Al-P (0.62), Al-V (0.58), and Ca-Mg (0.56), suggesting common origin from deposited PM and/or from material released from potentially damaged substrate. Strong correlations were also found between elements originating from fossil fuel combustion such as Ni-Zr (0.92), Ni-Cu (0.87), Ba-Zr (0.84), Cr-Zr (0.78), and Ni-V (0.62), suggesting inputs from resuspension of traffic-related road dust and/or direct emissions from combustion sources. Similar correlations were also previously found in particulate deposits from the Arch of Galerius in Thessaloniki (Samara et al. 2020). Interestingly, in the present study, high anticorrelation coefficients were observed between some elements such as Ca-Fe (−0.96), Ca-Si (−0.96), Ca-Al (−0.92), Ca-Mn (−0.91), Ca-V (−0.78), Fe-Mg (−0.56), and Al-Mg (−0.55). These anticorrelations are probably indicative of different mineral phases for these elements in deposition samples. An anticorrelation between Ca and Si is often in Saharan dust stemming from their differing origins and mineral forms within the dust plume (Varrica and Alaimo 2024).

### Ionic composition

Concentrations of ionic species in particulate deposits collected from monuments in Thessaloniki, Greece, and Nicosia, Cyprus, are provided in Table 2.

**Table 2.** Concentrations of ionic species in particulate deposits collected from monuments in Thessaloniki, Greece and Nicosia, Cyprus

Sample code	Cl^−^	NO_3_^−^	SO_4_^2−^	Acetate	Formate	Oxalate	Na^+^	K^+^	Mg^2+^	Ca^2+^	NH_4_^+^	Σ^−^/Σ^+^
Hagia Sophia Church												
DS1	4,314	6,292	6,256	5,709	1,253	ND	1,457	2,068	5,82	4,450	745	2.21
DS2	3,007	4,978	6,433	233	162	ND	3,338	2,331	494	5,122	1,279	1.26
DS3	404	680	2,026	92	ND	130	11,463	710	71	2,999	698	0.29
DS4	98	146	449	ND	107	ND	4,361	1,791	54	1,298	3,560	0.14
Mean	**1,956**	**3,024**	**3,791**	**1,509**	**381**	**32**	**5,155**	**1,725**	**300**	**3,467**	**1,571**	**0.91**
SD	**2,043**	**3,070**	**3,019**	**2,802**	**586**	**65**	**4,374**	**712**	**277**	**1,696**	**1,352**	**0.92**
Hamza Bey Mosque												
DB1	4,087	6,568	7,440	ND	ND	ND	1,018	1,324	753	10,052	ND	1.07
DB2	3,763	12,856	7,133	ND	ND	ND	9,595	2,477	812	14,088	ND	0.74
DB4	4,224	6,818	8,862	ND	ND	ND	3,510	1,740	1,148	15,461	ND	0.75
DB5	1,206	4,692	6,511	ND	ND	ND	6,545	1,178	414	8,699	ND	0.73
DB6	5,045	4,228	5,574	ND	ND	145	2,345	1,423	474	3,471	ND	1.76
DB7	1,143	3,066	1,156	ND	1563	ND	9,690	3,065	258	2,997	1,189	0.42
DB8	4,828	7,073	13,628	ND	ND	ND	6,587	9,160	1,233	27,179	ND	0.54
Mean	**3,471**	**6,472**	**7,186**	**-**	**223**	**21**	**5,613**	**2,909**	**728**	**11,707**	**170**	**0.85**
SD	**1,628**	**3,190**	**3,737**	**-**	**591**	**55**	**3,428**	**2,839**	**370**	**8,313**	**449**	**0.45**
Church of Archangel Michael Trypiotis												
DM2a	4,271	5,627	7,037	ND	ND	ND	3,140	482	1,250	12,331	2,972	0.78
DM3a	2,822	5,808	7,373	2,462	ND	ND	7,743	14,507	557	3,579	ND	0.85
DM3b	1,060	1,640	19,656	623	ND	ND	1,310	531	3,838	564	600	4.00
Mean	**2,718**	**4,358**	**11,355**	**1,028**	-	-	**4,064**	**5,173**	**1,882**	**5,491**	**1,191**	**1.78**
SD	**1,608**	**2,356**	**7,190**	**1,280**	**-**	**-**	**3,314**	**8,083**	**1,729**	**6,112**	**1,572**	**1.74**
Hadjigeorgakis Kornesios House												
DK4	2,737	3,744	21,336	ND	ND	ND	1,763	597	93	1,844	ND	7.88
DK5	6,069	12,051	22,868	ND	ND	ND	2,899	1,084	678	9,666	ND	2.59
DK8	2,356	2,901	24,515	ND	ND	ND	2,050	839	110	2,022	ND	7.59
DK11	4,702	4,169	24,856	ND	ND	ND	2,766	3,629	434	8,968	ND	2.32
DK12	1,228	245	3081	ND	ND	ND	8,46	347	195	1,608	ND	1.59
DK13	258	325	997	ND	ND	ND	40	294	106	1,325	ND	0.81
Mean	**2,892**	**3,906**	**16,276**	-	-	-	**1,727**	**1,132**	**269**	**4,239**	**-**	**3.80**
SD	**2,163**	**4,328**	**11,119**	**-**	**-**	**-**	**1,112**	**1,259**	**238**	**3,947**	**-**	**3.12**
Paphos Gate												
DP0	9,231	12,212	5,712	ND	ND	166	18,286	1,156	3,123	9,341	ND	0.74

*ND* non-detected

Sulfate appeared to be the prevalent anion in almost all deposit samples from Thessaloniki’s and Nicosia’s monuments excepting DB2 and DP0, in which nitrate clearly prevailed over sulfate. In certain samples, such as DB6, DK11, and DK12, chloride was the second most abundant anion exceeding nitrate.

Sulfate concentrations (449–13,628 μg/g and 997–24,856 μg/g in samples from Thessaloniki’s and Nicosia’s monuments respectively) were comparable with those previously found in particulate deposits from Hagia Sofia Church and the Galerius Arch (Papazachou & Samara 2013; Samara et al. 2020), and, in general, within the range of values reported in relevant literature (Fermo et al. 2015; McAlister et al. 2006 & 2008; Nava et al. 2010; Török et al. 2011). Nitrate concentrations (146–12,856 μg/g and 245–12,856 μg/g in samples from Thessaloniki’s and Nicosia’s monuments respectively) are well below those found in the Galerius Arch (3282–27,514 μg/g, Samara et al. 2020) and significantly lower than those reported for damaged layers of ancient ramparts in Salè, Morocco (> 50,000 μg/g), which were attributed to the immediate proximity of the monument to busy roads (Ozga et al. 2014). According to Fermo et al. (2018), considering their high solubility, nitrate concentrations on the marble surfaces can be affected by dissolution phenomena, and consequently, partial migration within the low porous microstructure of the specimens can occur. On the other hand, extremely high concentrations of NO_3_^−^ (> 90%) were found in salt blooms on damaged frescoes of S. Pietro a Corte in the historic center of Salerno (Campania, Italy) and were attributed to sewage leaking the frescoes (Ricciardi et al. 2022). Chloride levels ranged between 98 and 4,828 μg/g and between 258 and 9,231 μg/g in samples from Thessaloniki’s and Nicosia’s monuments, respectively. The presence of Cl^−^ in deposition samples is often attributed to marine aerosol and, in certain cases, to the use of anti-freeze salts in winter.

Acetate was the most abundant among organic anions in particulate deposits; however, it was detected only in samples from Hagia Sophia Church (92–5,709 μg/g) and the Church of Archangel Michael Trypiotis (ND–2,462 μg/g). These concentrations are significantly lower than those previously found in deposition samples from Galerius Arch in Thessaloniki (838–19,737 μg/g, Samara et al. 2020). Notably, acetic acid is an acid similar in pH to acid rain that can corrode many building materialsincluding marble, even less than hydrochloric or nitric acid (Sariisik & Sariisik 2011). Formate was quantifiable only in a few samples from Thessaloniki’s monuments (DS1, DS2, DB7) at concentrations relatively lower than those reported for Galerius Arch (107–1,563 μg/g). Lastly, oxalate was also quantifiable only in two samples from Thessaloniki’s monuments (DS3 and DB6) at concentrations 130 and 145 μg/g, respectively. Oxalates, often considered biological weathering products, are attributed to the effect of oxalic acid secreted from lichens or fungi on the carbonatic substrate; the calcium carbonate reprecipitates as calcium oxalate (Lamhasni et al. 2019). The general absence of oxalate in particulate deposits examined in this study agrees with previous data for the Arch of Galerius and the Hagia Sophia Church (Papazachou & Samara 2013; Samara et al. 2020).

Consistent with other studies (Fermo et al. 2015; Ozga et al. 2014), Ca^2+^ was the most prevalent cation ^+^(564–7,003 μg/g) in most samples, followed by Na^+^ (40–4,798 μg/g). However, Na^+^ exceeded Ca^2+^ in some samples (DS3, DS4, DB7, DM3a, DM3b, DP0). K^+^ concentrations ranged between 140 and 11,851 μg/g, with the highest value found in DB8 collected from a limestone substrate rich in K_2_O. NH_4_^+^ was detectable only in the four samples from the Hagia Sofia Church (698–3,560 μg/g), one sample from the Hamza Bey Mosque (1,189 μg/g), and two samples from the Michael Trypiotis Church (600 and 2,972 μg/g). The presence of NH_4_^+^ could be attributed to deposition of ammonium sulfate and ammonium nitrate aerosols that are secondarily formed through the reaction of sulfuric and nitric acids with atmospheric ammonia (Pozo-Antonio et al. 2022).

Correlations between ionic species are shown in Table S2.

As can be seen, no correlation was found between SO_4_^−2^ and Ca^2+^ that could imply sulfation of calcite; on the contrary, significant correlations were found for NO_3_^−^ with Ca^2+^ and Mg^2+^ that indicate a degradation effect of NO_*x*_ resulting in the formation of calcium nitrate and magnesium nitrate. Magnesium nitrate has also been identified as a degradation product in other studies highlighting the fact that new formation salts represent a worrying phenomenon since these salts can induce the formation of microcracks inside the stone because of changes in volumes (Fermo et al. 2015). Significant correlations in this study were also found for Cl^−^ with Ca^2+^ and Mg^2+^ indicating the co-existence of crustal dust and marine aerosols, or secondary anthropogenic pollutants often pointing to the chemical reaction between sea salt and calcium-rich dust. The correlation between Na^+^ and K^+^ typically indicates a shared source of emission, most commonly originating from marine aerosols, biomass burning, or industrial activities. The correlation between NO_3_^−^ and K^+^ also supports the contribution of biomass burning that is a significant source of fine particles in the atmosphere of Thessaloniki (Argyropoulos et al. 2017). No considerable correlations were found for organic anions similarly to Samara et al. 2020.

Stoichiometric ratios of the concentrations of ionic species are often used in airborne PM studies to estimate the relative contribution of marine sources. In the present study, two ratios were calculated and compared to the reference values for sea salt, [Cl^−^]/[Na^+^] and [SO_4_^2−^]/[Na^+^] Only few deposition samples from Thessaloniki’s monuments (DS3, DS4, DB7) exhibited [Cl^−^]/[Na^+^] ratio values lower than 1.8, which is the reference value for sea salt, indicating potential Na^+^ sources other than marine spray, e.g., resuspended dust (Fermo et al. 2018). On the contrary, most samples from Thessaloniki and all samples from Nicosia exhibited [Cl^−^]/[Na^+^] ratio values > 1.8 (up to 4.0 and 6.4, respectively) suggesting potential marine origin for Na^+^. The [Cl^−^]/[Na^+^] values found in deposition samples from Thessaloniki’s monuments are in agreement with the corresponding ratio values found in airborne PM_2.5_ that ranged from ≤ 1.8 at an urban background site to > 1.8 in the city center (Voutsa et al. 2014). Additionally, the value of the [SO_4_^2−^]/[Na^+^] ratio was higher than the reference value for sea salt (i.e., 0.25) in most samples of particulate deposits from Thessaloniki’s monuments (up to 7.3 in DB1), and in all samples from Nicosia’s monuments (up to 24 in DK13) suggesting a predominantly anthropogenic origin for sulfates (Fermo et al. 2018). A [SO_4_^2−^]/[Na^+^] ratio value of 20 has been reported for deposits on marble in Fermo et al. (2018).

Nonetheless, ionic ratios should be used with great caution in particulate deposit samples because they depend not only on the size of particles deposited and the atmospheric conditions, but, primarily, on the intrinsic activity of the deposition surfaces; therefore, it is not easy to distinguish between atmospheric deposition and other sources of soluble salts. Kloppmann et al. (2014), examining the sources and mechanisms of the uptake of soluble salts from porous limestone medieval statues in SE France, concluded that the effects of air pollution are detectable but appear less significant than other sources of soluble salts, such as gypsum restorations that contribute locally to the neoformation of sulfates, and the presence of sewage or organic fertilizers that contribute to nitrates.

Decay of monument materials depends on acidic dry deposition of particles. There are no literature data concerning the acidity of deposition samples from monument surfaces. However, given the large size of these particles that probably contain dust, sea spray, and biomass burning emissions, their acidity is expected to be lower than those estimated for the fine aerosol in the Eastern Mediterranean where high sulfate and nitrate levels, the main drivers of acidity, were predicted in other studies (Kakavas et al. 2021; Neroladaki et al. 2025).

### FT-IR analysis results

**Fig. 4:**
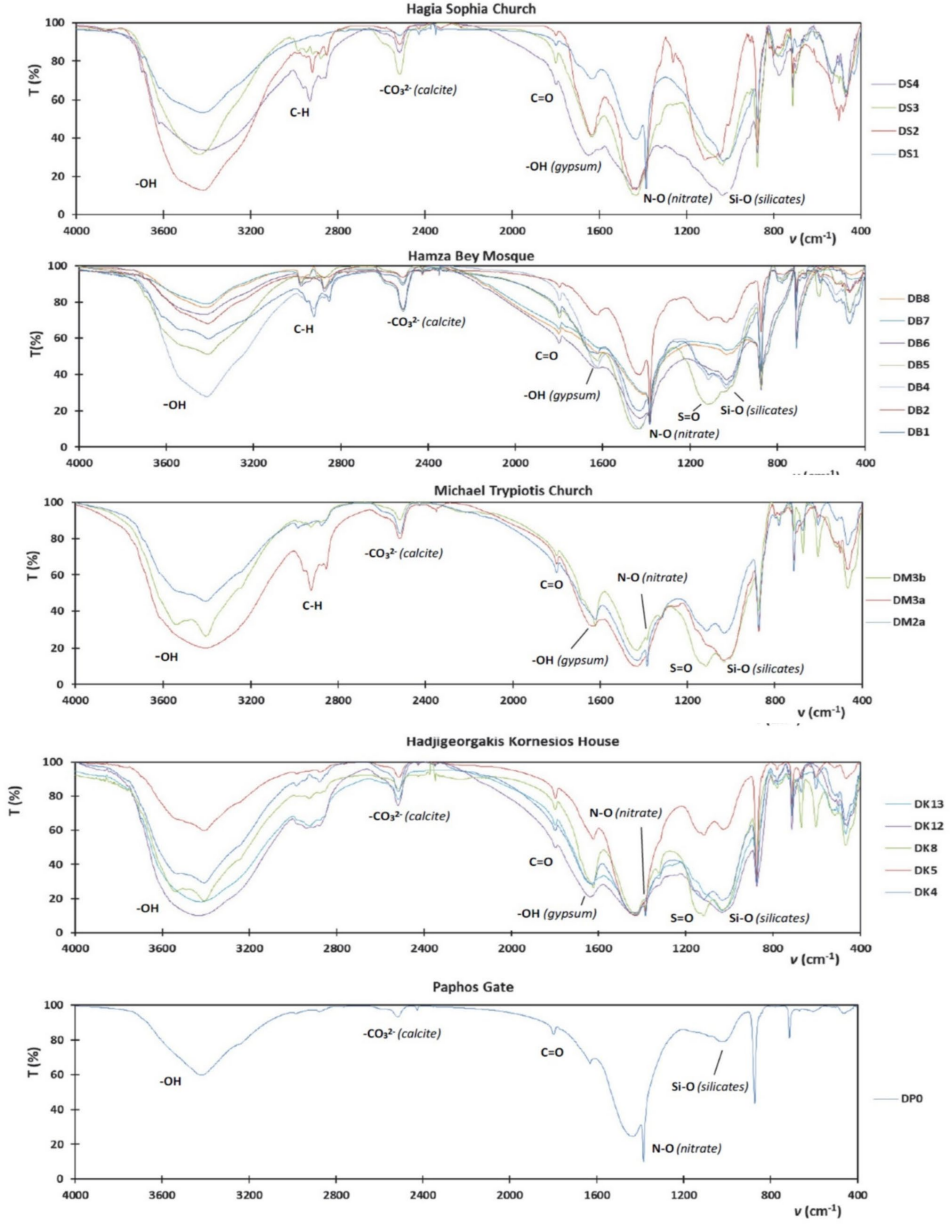
FTIR Spectra of the particulate depositions samples from the studied monuments in Thessaloniki, Greece, and Nicosia, Cyprus (sample codes as in Table 1)

The FTIR spectra of the particulate deposition samples from the studied monuments are shown in Fig. 4.

Apparently, all samples presented quite similar absorption spectra. Given that most of the sampling substrates consisted of marble and limestone, calcite was identified in all samples by the characteristic absorption peaks at 2518–2515, 1802–1759, and 1448–1439, presented simultaneously with the ones of carbonates (Fermo et al. 2018, La Russa, et al. 2009; Maravelaki-Kalaitzaki 2005). A peak at 1000–1026 cm^−1^ was also evidenced in all samples indicating the presence of silicates (La Russa et al. 2009; Lamhasni et al. 2019). Calcite is a natural component of calcarenite, while silicates may be natural components or originating from wind-born particles incrustations (Maravelaki-Kalaitzaki 2005). Gypsum was identified through the absorption bands assigned to O–H functional groups (the broad and intense peaks at 3531–3425 cm^−1^) followed by peaks at 1692 and 1624–1615 cm^−1^, due to water within the contained gypsum (CaSO_4_·2H_2_O) presented into the broad (1105–1101), and weak (597–607, 598, 460–454 cm^−1^) vibrations of SO_2_ (La Russa et al. 2009 & 2013; Fermo et al. 2018; Varrica et al. 2019). Gypsum was clearly detected in relatively few deposition samples (DB5, DK8, DM2a, and DM3b), the same that also exhibited high S content in XRF analysis. Aliphatic hydrocarbons were clearly identified mostly in samples DS2, DS4, DB1, DB6, DB7, DB8, and DM3a due to the sharp, medium-intensity peaks in the area 2852–2920 cm^–1^ (La Russa et al. 2009). No firm evidence for aromatic hydrocarbons (C=C) was shown. The strong absorbance band in the 1650–1750 cm^−1^ is attributed to stretching vibrations of C=O existing due to carbonyl groups (e.g., ketones, esters, carboxylic acids). The small peaks in the region 1750–1820 cm^−1^, found almost in all samples, can be attributed to the carboxyl group (e.g., fatty acids, amino acids) as symmetric and asymmetric stretch of C=O (La Russa, et al. 2009). Finally, a small peak (almost a shoulder) at 1385 cm^−1^, indicative of the presence of nitrate (Fermo et al. 2018; Varrica et al. 2019), was shown in several samples (DS1, DB1, DB2, DM2a, DK5, DK8, DK11, DK13). This peak is more pronounced in sample DP0.

### Py-GC/MS analysis results

**Fig. 5:**
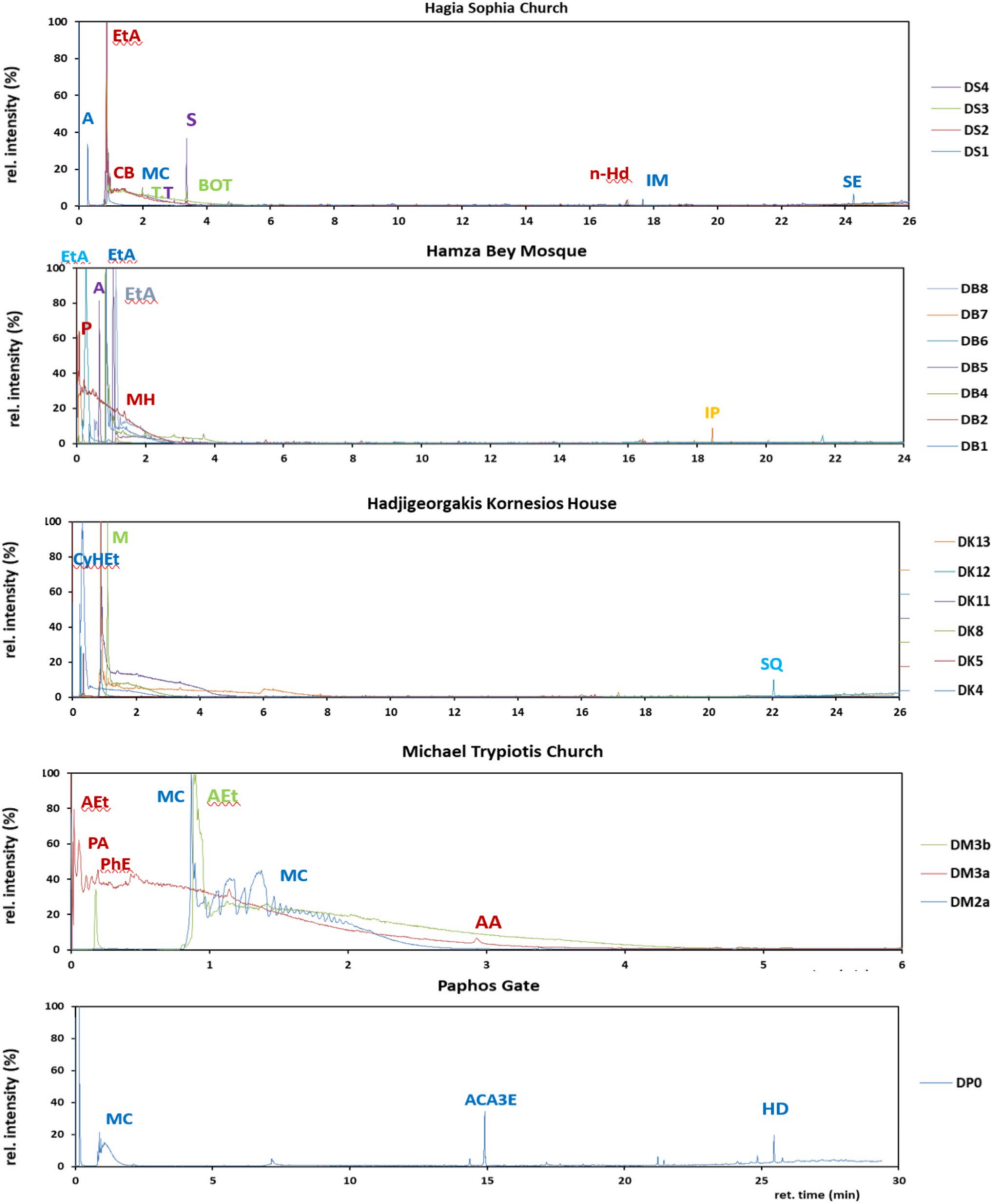
Pyr-GC/MS spectra of deposition samples from the studied monuments (Α, argon; AA, acetic acid; ACA3E, o-acetylcitric acid triethyl ester; AEt, l-alanine ethylamide (S)-; BOT, bicyclo[4.2.0]octa-1,3,5-triene; CB, cyclobutane; CyHEt, (S)-(+)−1-cyclohexylethylamine; EtA, ethylamine; HD, cis-9-hexadecenal; IM, isopropyl myristate; IP, isopropyl palmitate; MC, carbamic acid monoammonium salt; MH, heptane-3-methylene; n-Hd, n-hexadecanoic acid; P, 4-Penten-1-ol; PA, pentanal; PhE, phenylephrine; PM, 2-propanamine; 1-methoxy; S, styrene; SE, supraene; SQ, squalene; T, toluene)

The pyrograms of particulate deposition samples are shown in Fig. 5.

In addition, the relative abundances of the detected pyrolysis products are provided in Tables S3a and S3b.

Both qualitative and quantitative differences in the distribution of pyrolysis products have been found among samples with most fragments being gathered in 0–5 min. Samples from the Church of Hagia Sophia exhibited different pyrolysis fingerprints. The most abundant pyrolysis product in DS1 was carbamic acid monoammonium salt; in the pyrolysate of DS2, the dominant compound was ethylamine followed by lower abundances of cyclobutanol, DS3 was dominated by 1,2-propanediamine, while (S)-L-alanine-ethylamide and styrene were the most abundant compounds in DS4. Ethylamine was the most abundant pyrolysis product in samples from the Hamza Bey Mosque (DB1, DB6, and DB8), while other samples from the same monument exhibited presence of other organic macromolecules such as 4-Penten-1-ol, 1,2-Propanediamine, n-hexadecanoic acid, and cyclopropylcarbinol in DB2, and isopropyl palmitate (an ester of isopropyl alcohol and palmitic acid) in DB7. 1-methoxy-2-propanamine and l-alanine ethylamide, (S)- fully dominated DB5, whereas methyl-hydrazine and 3-methyl butanal were detected in DB4, collected from a metallic substrate. Traces of naphthalene (not shown in Table S3a) were also detected in DB4 in agreement to Sýkorová et al. (2011), who detected naphthalene, methylnaphtalenes, and phenanthrenes in pyrolysates of black layers from the Prague Castle.

Regarding samples from the Archangel Michael Trypiotis Church, the pyrolysate of DM2a presented mainly carbamic acid monoammonium salt. Pentanal was detected in the pyrolysate of DM3a, while (S)-L-alanine-ethylamide dominated the pyrolysate of DM3b. In samples from Hadjigeorgakis Kornesios House, (S)-(+)−1-cyclohexylethylamine fully dominated the pyrolysate of DK4, followed by isopropyl myristate in the pyrolysate of DK5. Carbamic acid monoammonium salt and 1-methyldodecylamine were major pyrolysis products in DK8 and DK11, respectively. (S)-L-alanine-ethylamide dominated the pyrolysate of DK12, followed by lower abundances of squalene. Finally, acetic acid and cyclopropyl carbinol were detected in the pyrolysate of DK13. As for the Paphos Gate sample (DP0), the dominant pyrolysis product was carbamic acid monoammonium salt with lower abundances of (S)-L-alanine-ethylamide and 2-amino-heptane.

Although it is difficult to distinguish the sources of all the high-molecular-weight organic compounds detected in the pyrolysates of deposition samples because certain compounds may not be pyrolysis products but simply survive the harsh pyrolysis conditions, the pyrolysis products results can be important to the environmental conditions in this study. For instance, carbamic acid monoammonium salt can be a pyrolysis product, particularly from the thermal decomposition of nitrogen-containing compounds like urea or nitrogen-containing biomass. In addition, it has reported several uses (cosmetic, domestic, commercial in biocidal or pesticide products, etc.), while it is a component in processes of exhaust emission denitrification (US Epa 2006). Ethylamine can be produced via the pyrolysis of amino acids and amines, such as alanine and diethylamine. (S)-L-alanine-ethylamide can be a pyrolysis product, particularly when L-alanine or related compounds are subjected to thermal decomposition, especially in the presence of ethylamine or under conditions where ethylamine is generated. Hexadecanoic acid (palmitic acid) is a major component of atmospheric organic aerosol in urban areas since it is one of the dominant chemical species in fresh gasoline emissions (Lui et al. 2023). Moreover, it can be produced from the pyrolysis of certain organic compounds, particularly those containing fatty acids or lipids. Acetic acid can be produced through the pyrolysis of lignocellulosic biomass. Squalene, a natural triterpene, present in vegetable oils, as well as in human skin oil and certain personal care products, can be produced through the pyrolysis of organic compounds deriving from biomass like algae or sunflower oil. (S)-(+)−1-cyclohexylethylamine, used in pharmaceutical and agrochemical applications, is not a typical pyrolysis product of organic materials. PAHs are typical combustion products being deposited on the surface of buildings from the atmosphere; moreover, they can be formed during biomass pyrolysis. Styrene has been found in pyrolyzates of black crusts (Sýkorová et al. 2011); furthermore, it is major pyrolysis product of ambient PM10 along with benzene, toluene, and 1-undecene (Chae and Choi 2022) that were also detected in some deposition samples in this study. However, styrene can also be a product of pyrolysis of proteins, peptides, lignins, or tannins (Sýkorová et al. 2011). The toluene/styrene ratio in pyrolysates can be used as an indicator of the presence of synthetic polymers (Dignac et al. 2005). If this ratio is greater than 1, styrene is considered a pyrolysis product of natural substances. In our study, styrene that was found only in one sample from the Hagia Sofia Church (DS4) predominated slightly over toluene (toluene/styrene ratio 0.95); thus, its presence could be rather attributed to synthetic polymer residues (tire and asphalt wear particles, and plastics such as polystyrene, polypropylene, or polyethylene terephthalate) (Chae and Choi 2022).

The pyrolysis fingerprints of the particulate deposition samples in this study are different from those of the black crusts from the Prague Castle, where benzonitrile, benzene, toluene, naphthalene, biphenyl, dibenzofuran, and quinoline were the major pyrolysis products, and styrene (as a pyrolysis product of synthetic polymers) was found in all samples as the second most abundant compound (Sýkorová et al. 2011).

## Conclusions

A multi-analytical approach including non-destructive and destructive techniques was applied to the particulate depositions (< 100 μm), collected from various surfaces of monuments located at urban sites in the cities of Thessaloniki (Greece) and Nicosia (Cyprus), focusing on a detailed analysis of their morphology and chemical composition.

The deposition samples exhibited quite similar geochemical compositions consisting of CaO, SiO_2_, Al_2_O_3_, and Fe_2_O_3_, which indicates impact from road dust and/or Saharan dust. The impact of road dust was more evident in Thessaloniki monuments where concentrations of crustal elements (Ca, Fe, Si, Al, and Ti) were higher at low-height sampling points in comparison to the more elevated points. Moreover, the effect of traffic was evident on sides of monuments oriented towards busy roads with higher concentrations of anthropogenic elements such as Zn, Ba, Cu, V, Ni, Zr. Elements such as Ba, used in previous consolidation works, and Pb, used as a protective coating on the iron connections of stone blocks, were identified in some samples. NaCl was detected in samples from the Hamza Bey Mosque in Thessaloniki, as well as the Hadjigeorgakis Kornesios House and the Paphos Gate in Nicosia, indicating an effect of marine aerosol. In addition to limestone and silicates, the FTIR analysis indicated in all monuments the presence of organic compounds including aliphatic hydrocarbons, as well as carbonyl- and carboxyl-containing compounds. Peaks, indicative of the presence of gypsum, were observed only in a few samples, one in Thessaloniki and three in Nicosia. Finally, nitrates were detected in many samples from the monuments studied in both cities. Pyrolysis-GC/MS analysis showed a wide spectrum of high-molecular-weight organic substances at variable abundances. Carbamic acid monoammonium salt and (S)-L-alanine ethylamide were the major pyrolysis products found in almost all samples. Other organic substances with lower frequency of detection included ethylamine, 1,2-propanediamine, 1-methoxy-2-propanamine, 1-methyldodecylamine, (S)-(+)−1-cyclohexylethylamine, hydrazine, acetic acid, styrene, and PAHs.

Conclusively, this study revealed that the studied urban monuments are affected by both natural and anthropogenic sources, including local and/or transported dust, traffic-related pollutants, residues of plant and animal organisms and microorganisms, biomass residues, and synthetic polymer residues. The results may provide useful information to local authorities for more effective protection and preservation of the architectural heritage in the two Mediterranean countries. Furthermore, the periodical and long-term monitoring of deposited particles could be useful for better understanding the effects of the surrounding environment and anthropogenic activities on urban monuments.

## Supplementary Information

Below is the link to the electronic supplementary material.

ESM 1 (DOCX 11.6 MB)

## Data Availability

Data is available upon request.
