# Supplementary material for: Characterization of particulate depositions collected from archeological monuments in Greece and Cyprus using multiple analytical techniques
Source: Environ Sci Pollut Res Int. 2026 May 15;33(17):8276–93. doi: 10.1007/s11356-026-37793-x (PMC13226405; doi:10.1007/s11356-026-37793-x)
Supplement: Supplementary file 1 — (DOCX 11.6 MB) [file 11356_2026_37793_MOESM1_ESM.docx]

**Characterization of particulate depositions collected from archaeological monuments in Greece and Cyprus using multiple analytical techniques**

**Thaleia Gkraikou^1,4^, Argyri Kozari^1^, Evangelia Vouvoudi^2^, Lambrini Papadopoulou^3^, Vasilios Melfos^3^, Brunella Santarelli^4^, Constantini Samara^1*^**

*^1^ Aristotle University of Thessaloniki, Department of Chemistry, Laboratory of Environmental Pollution Control, 54124 Thessaloniki, Greece*

*^2^ Aristotle University of Thessaloniki, Department of Chemistry, Laboratory of Polymers and Colours Chemistry and Technology, 54124 Thessaloniki, Greece*

*^3^ Aristotle University of Thessaloniki, Department of Geology, Section of Mineralogy-Petrology-Economic Geology, 54124 Thessaloniki, Greece*

*^4^ The Cyprus Institute, Science and Technology in Archaeology and Culture Research Center (STARC),* *Konstantinou Kavafi 20, Aglantzia 2121, Nicosia, Cyprus*

**Description of sites and monuments**

A map showing the sampling sites and the monuments is provided below.


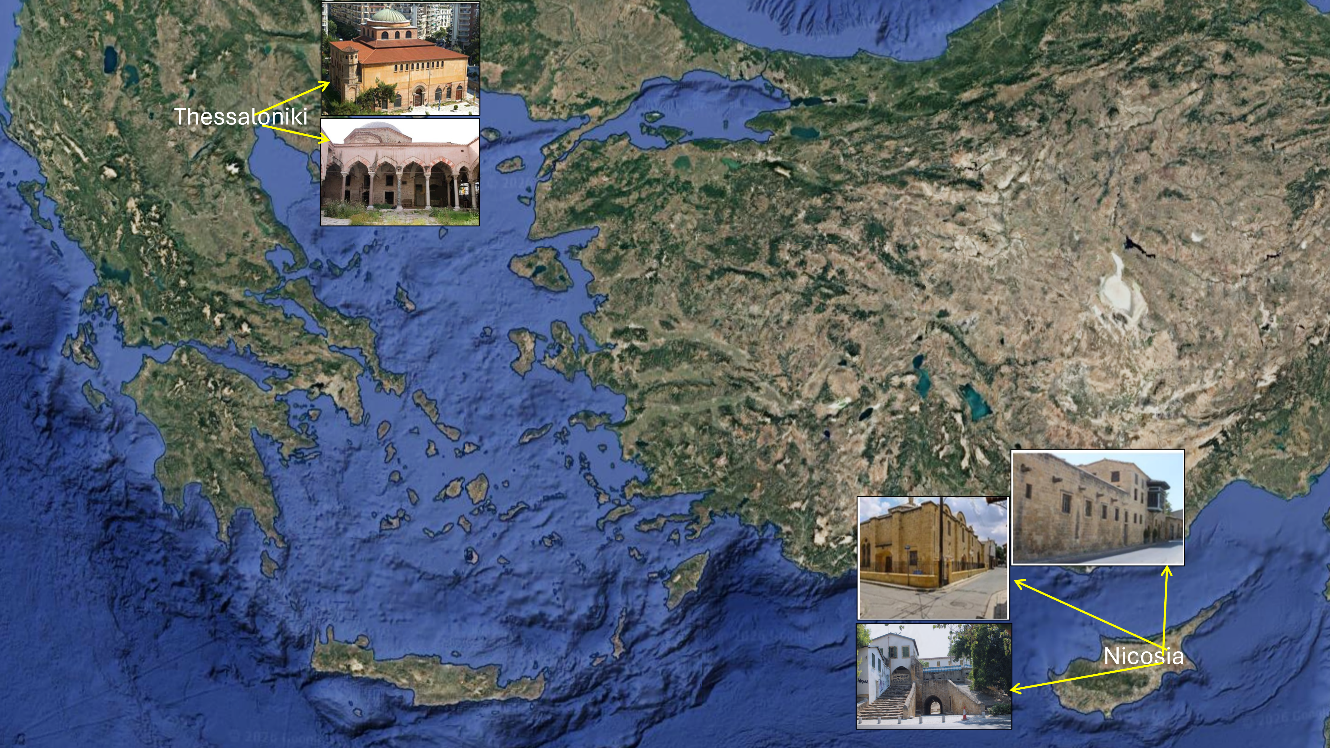


**Map showing the sampling sites and the monuments**

The monuments sampled in Thessaloniki, Greece, were the church of Hagia Sophia and the mosque of Hamza Bey. The church of Hagia Sophia is an early Christian monument built in the 7^th^ century on the site of a church destroyed from an earthquake in 620 AD (Toumpouri 2022). The architectonic type is transitional cross-in-square. Multiple interventions occurred; first due to an earthquake in the 9^th^ c., then an addition of a narthex at the 10^th^ c., the transition to a Muslim temple at 1430 during the Ottoman occupation and last the return to the Christians at 1913 after being destroyed from a fire in 1890. The latest consolidation and restoration work achieved on the monument followed the disastrous 1978 earthquake. Because of its outstanding Byzantine art and architecture, in addition to its importance in early Christianity, it is one of [several monuments in Thessaloniki](https://en.wikipedia.org/wiki/Paleochristian_and_Byzantine_monuments_of_Thessaloniki) listed as a [UNESCO](https://en.wikipedia.org/wiki/UNESCO) [World Heritage Site](https://en.wikipedia.org/wiki/World_Heritage_Site) in 1988. The monument is in the center of Thessaloniki, located at a lower ground level than the surrounding streets that are usually characterized by high-volume traffic. The Hamza Bey Mosque is a 15th-century Ottoman Mosque built in 1468. The monument, located 800 m northwest of the church of Hagia Sophia, is the only building of such architectonic type in the Balkans, being similar to the mosques of Adrianople and Constantinople in Turkey (Raptis 2012; Mohamed 2022). Many interventions took place during the 16^th^ c., and many of its construction materials are in second use, deriving from buildings of the early Byzantine Thessaloniki.

The monuments sampled in Nicosia, Cyprus were the church of Archangel Michael Trypiotis, the Hadjigeorgakis Kornesios House, and the Paphos Gate. The Archangel Michael Trypiotis Church is a Greek Orthodox Church in the old town of Nicosia, Cyprus funded by offerings from believers in 1695. The church is known for its decorated iconostasis, the largest within the walls of Nicosia, which was carved in 1812 and gilded in 1816. The church houses the relics of the Bishop of Rigaina, the miraculous icon of Archangel Michael (1634) and other icons by renowned painters of the 17^th^ and 19^th^ c. The Hadjigeorgakis Kornesios House, located 550 m south-easterly of the Archangel Michael Trypiotis church, is the most important surviving urban architecture building from the end of the 18^th^ century. The building material is local poros stone (marly limestone and sandstone). Above the entrance there is a built-in marble slab with a relief depiction of the winged lion of Venice standing on a Gospel with Latin writing and a cross. A relief plaque bears the date 1793 and is located on the inner side of the front door. Another marble, a courtyard plaque dated from 1803, decorates the fountain, where water flowed into a rectangular basin, an ancient sarcophagus in second use. Finally, the Paphos Gate is one of the three entrances to the Venetian walls surrounding Nicosia and was named after the southwest city where the gate leads, Paphos. The gate consists of an opening in the wall with vaulted roof. The gate was constructed by second-hand materials originating from earlier buildings in the area during medieval times (Artopoulos et al. 2018).

***Literature***

Artopoulos G, Gregoriou C, Ioannou C (2018) Immersive Design Practices, from Virtual to Real Space and Back–The Case of Reactivating Paphos Gate. 4th Biennial of Architectural and Urban Restoration, Nicosia, Cyprus, July 2018.

Mohamed A (2022) Uncommon Layouts in the Plans of Ottoman Mosques in Greece (Rohodes and Crete Islands). مجلةکلیةالآثار . جامعةالقاهرة, 12(2022), 361–390.

Raptis KT (2012) Early Christian and Byzantine ceramic production workshops in Greece: typology and distribution. Atti del IX Congresso Internazionale sulla Ceramica Medievale nel Mediterraneo (Venezia 23-27 Novembre 2009), Issue February, 38–43.

Toumpouri M (2022) Hagia Sophia of Thessaloniki. [UBC Library Open Collections](https://open.library.ubc.ca/media/download/pdf/52387/1.0422127/2), 12 July 2022, p. 1–22.

**Methods of sample characterization and analysis**

The SEM setup (Zeiss EVO 15) coupled with an advanced (Oxford Aztec) energy dispersive spectrometer (EDS) with a sampling depth of 1-2 µm, capable to detect elements from C to U in amounts as low as 1.0% wt., was used for sample imaging and microanalysis of individual particles. The sample surface had to be conductive, and for that a Quorum Q 150RES Plus carbonization device using Au (for magnification up to ×50k) was employed. Briefly, using a stereo microscope (Zeiss Stemi 305), a small amount of the sample was placed upon aluminum specimen stubs (Agar Scientific) with adhesive surface (diameter 12 mm carbon tabs of Agar Scientific). The sample was placed over the adhesive surface and residues were removed using a rubber blower (Anchor Brand). All the tools (forceps etc.) were cleaned with isopropanol solution to avoid contamination.

The X-Ray Fluorescence (XRF) analysis, providing elemental information averaged over a larger sample volume in comparison to EDS, was employed to determine the bulk chemical composition of the samples. The XRF analysis was performed using two different devices: (a) a fixed type S4-Pioneer (Bruker-AMS, Deutschland) following fusing of the samples with a mixture of borates to prepare a vitrified tablet, and (b) with a Hitachi XMET 8000 portable XRF requiring no sample preparation. To prepare the samples, 8-8.5 g of Li_2_B_4_O_7_ was weighed and then ca. 1–0.5 g of the sample was added, along with a drop of liquid LiI.

The concentrations of inorganic and organic anions (chlorides, sulfates, nitrates, acetates, formates, and oxalates) and cations (sodium, potassium, calcium, magnesium) were determined by Ion Chromatography (IC-CD, Shimadzu LC-10ADVP) according to previously published procedures (Papazachou & Samara 2013; Voliotis et al. 2017; Voutsa et al. 2014; Samara et al., 2020). Briefly, a sample quantity of 0.1 g was extracted with 2.5 mL of ultrapure water in an ultrasonic bath for 30 min. The extractants were filtered through a 0.45 mm membrane and stored in the refrigerator until analysis. Anions were separated on a Slimpack (Shimadzu, model IC-A1) column using potassium hydrogen phthalate as mobile phase. Cations were separated on a Universal Cation (Alltech, model 7u) column using methane sulfonic acid as mobile phase. Column temperature was kept at 40 °C and the temperature of the detector cell was 43 °C. Flow rate was 1.5 mL/min and injection volume 100 mL.

The FT-IR analysis was performed in a Spectrum One spectrometer (Perkin Elmer, USA), equipped with the Spectrum v.3.1 software, using the KBr disk analytical technique. The scanning range of this spectroscope was 4000 – 400 cm^-1^ with resolution 4 cm^-1^. The number of scans was 16. A small amount of sample (~2 mg) was mixed with 180 mg of KBr (Sigma Aldrich^®^) in a porcelain mortar and grounded to a uniform powder, which was then compressed under high pressure (15 tons for 5 min) to form a transparent disc. A KBr disc was used as a blank. Samples To mitigate moisture absorption, the KBr powder and the samples were dried before use, and the discs were prepared in a dry environment. The evaluation of IR spectra was done with the help of absorption data tables from 4000 to 600 cm-1 for the identification of the characteristic groups of the main peaks. The region below 1000 cm^-1^ is called the fingerprint area and is relatively difficult to evaluate accurately alone.

Pyrolysis gas chromatography/mass spectrometry (Py-GC/MS) was employed for the identification of organic macromolecules. A non-polar glass capillary column consisting of 95% dimethylsiloxane (30 m × 0.25 mm *(i.d*) × 0.25 μm) MEGA-5HT (Column ID#171,734, Italy) was used for chromatographic separation. A tiny quantity (~20 mg) of each sample was placed directly into cylindrical stainless-steel cups and pyrolysed for 0.3 min at the temperature of 700 ^o^C, chosen after some trials in the range 300-750 ^o^C. Chromatographic analysis was carried out with a temperature program ranging from the initial temperature 50 °C for 1 min to 300 °C with a heating rate10 °C/min, and final constant temperature for 1 min. The total program time was set at 27 min and the interface temperature at 300 °C. The ion source operated at 200 °C, the mass range detected was from 40 to 600 amu, the data acquisition rate was 0.1 s and the detector voltage was 1.02 kV. He was circulated with a continuous column flow of 1 mL/min and purge flow of 3 mL/min, total flow at 104 mL/min, inlet pressure at 53.4 kPa, linear velocity at 36.3 cm/s and split ratio at 1:50. The GC/MS post-run analysis software was used for chromatogram processing. The NIST library (Shimadzu 2011) was used for peak identification. The results after chromatogram processing were gathered a few peaks, in terms of intensity (A/H ratios).

| **DS1** | 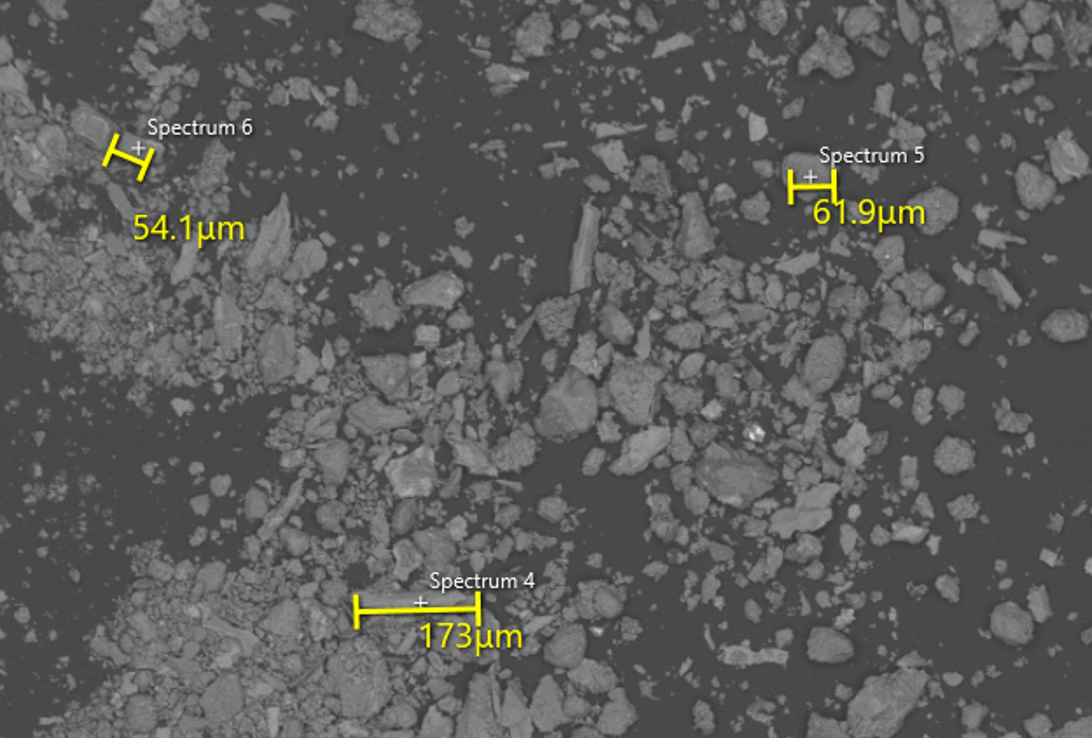 | **DS2** | 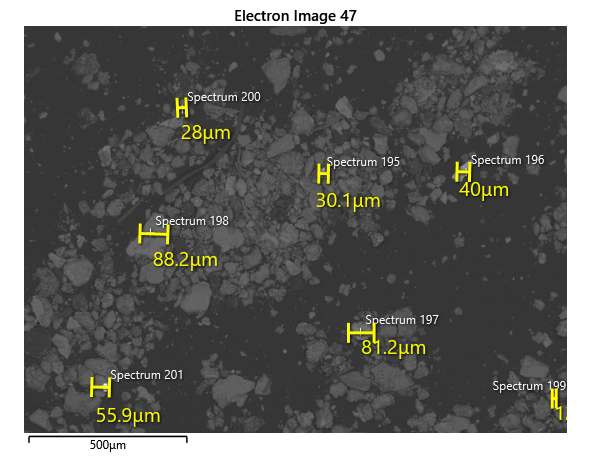 |
| --- | --- | --- | --- |
|  |  |  |  |
| **DS3** | 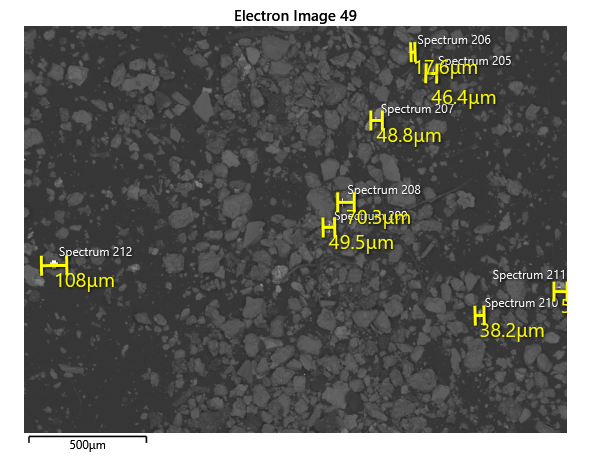 | **DS4** | 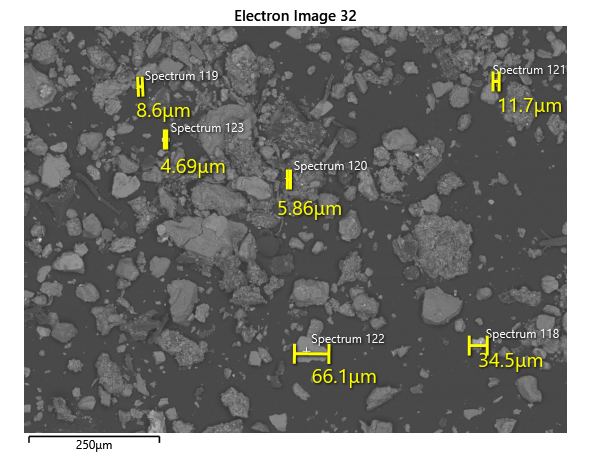 |
|  |  |  |  |
| **DB1** | 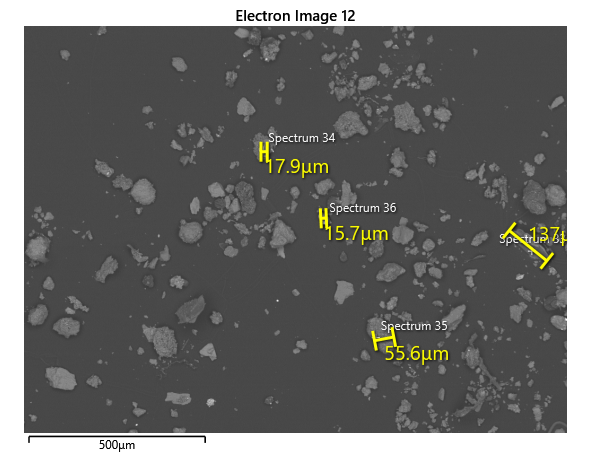 | **DB2** | 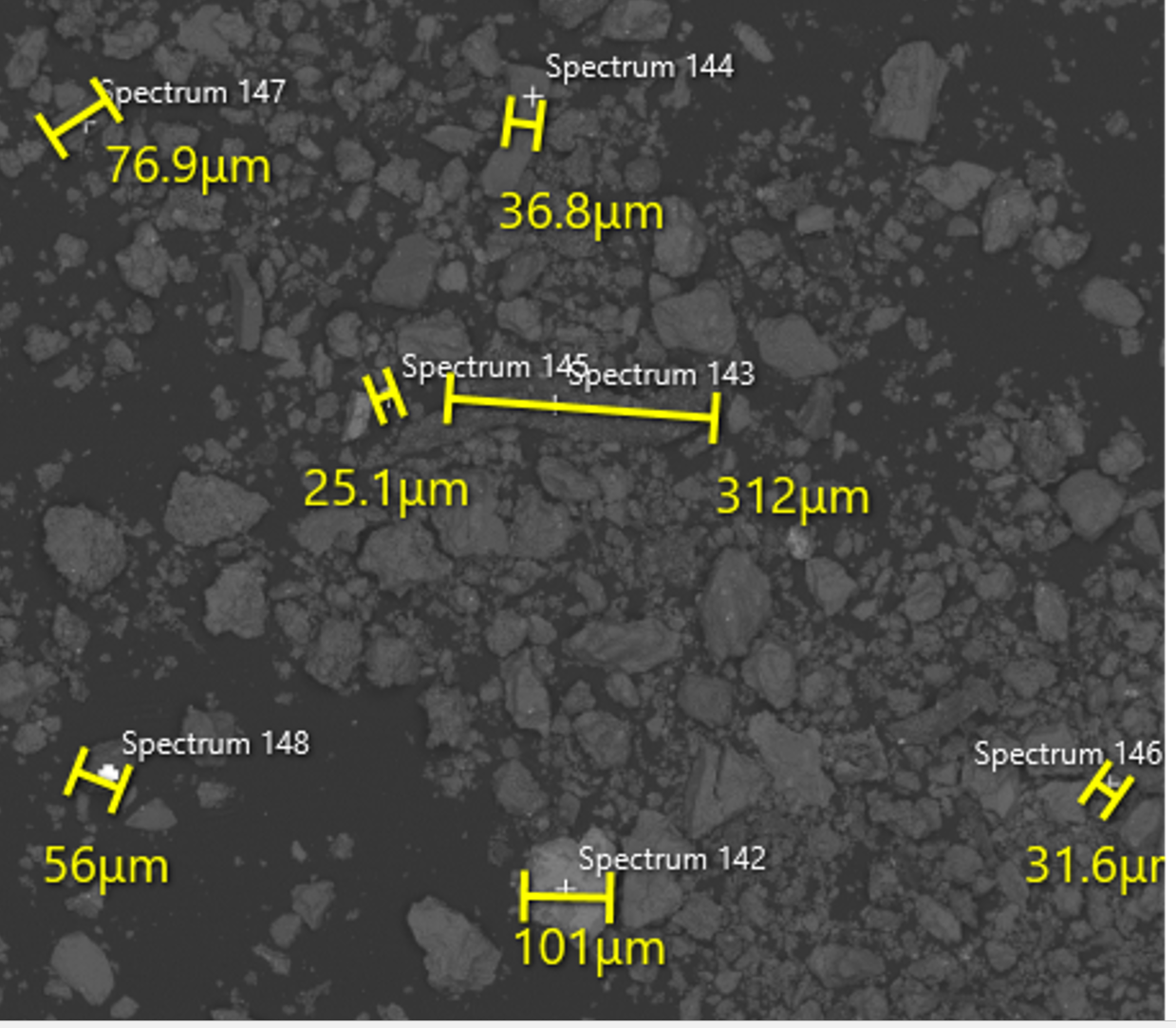 |
|  |  |  |  |
| **DB4** | 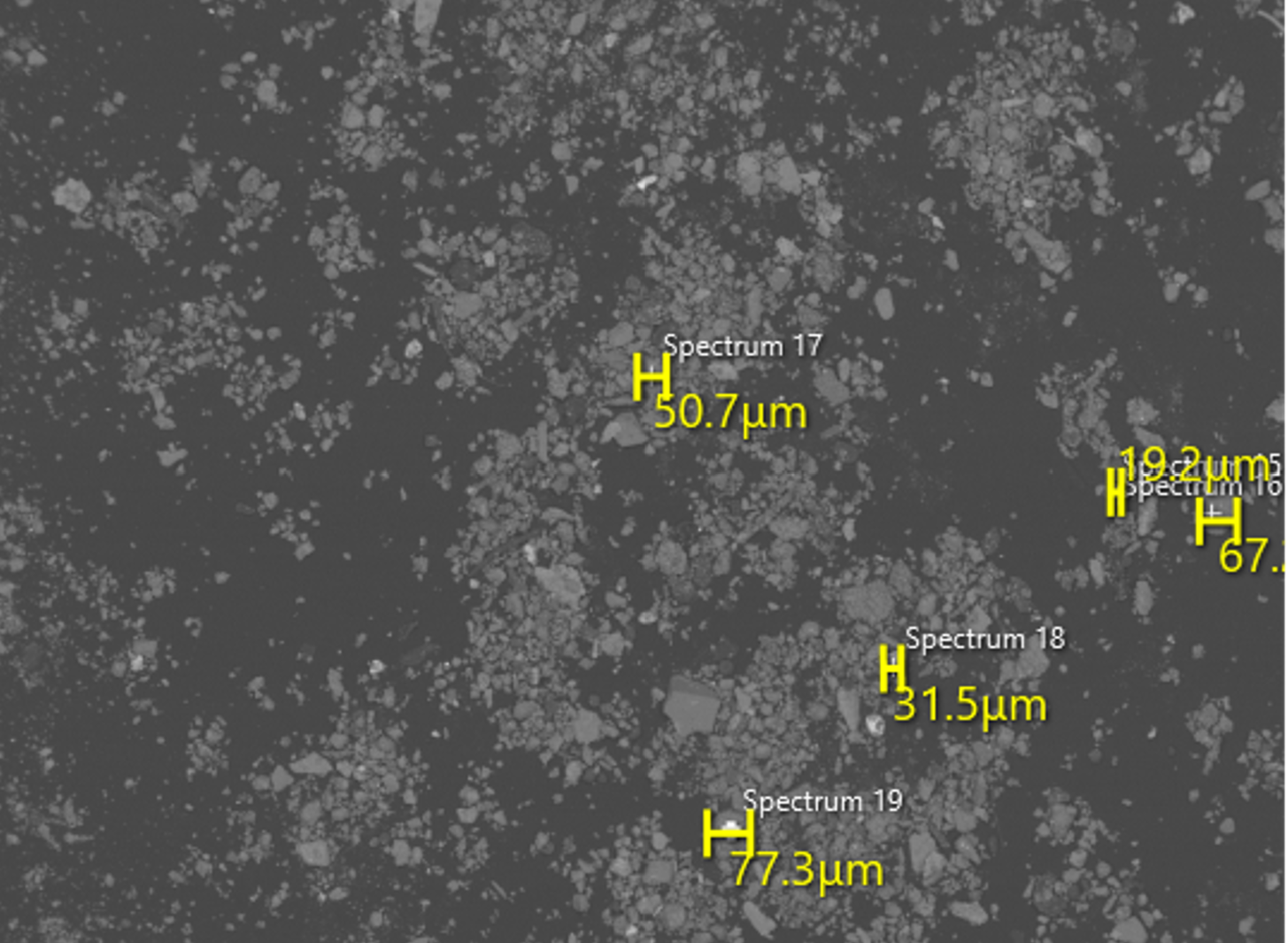 | **DB5** | 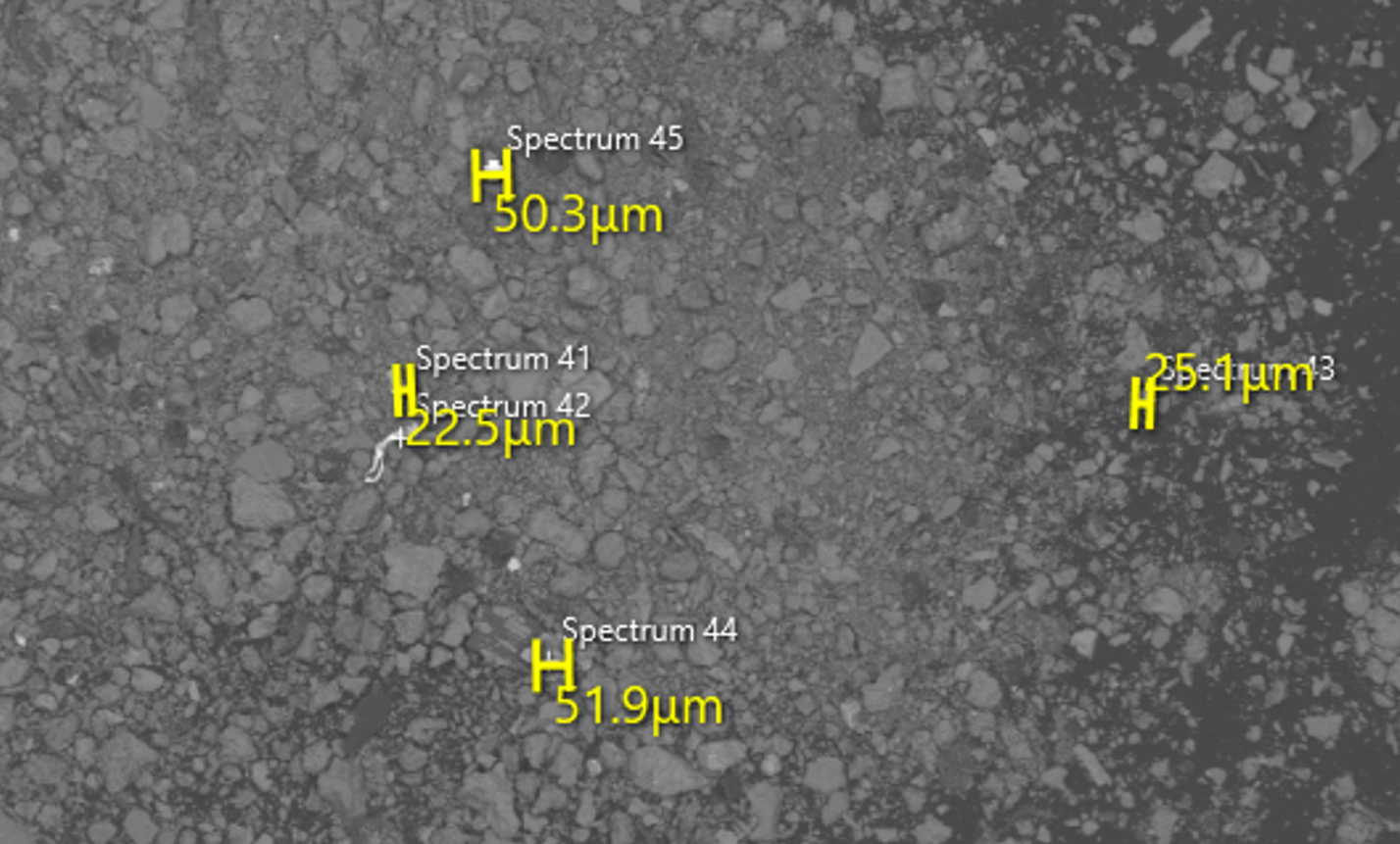 |
|  |  |  |  |
| **DB6** | 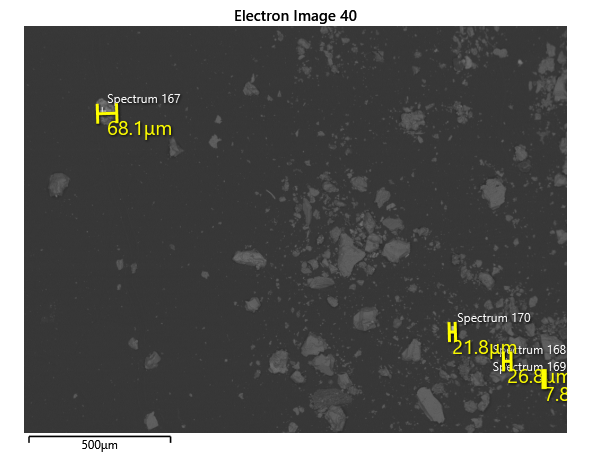 | **DB7** | 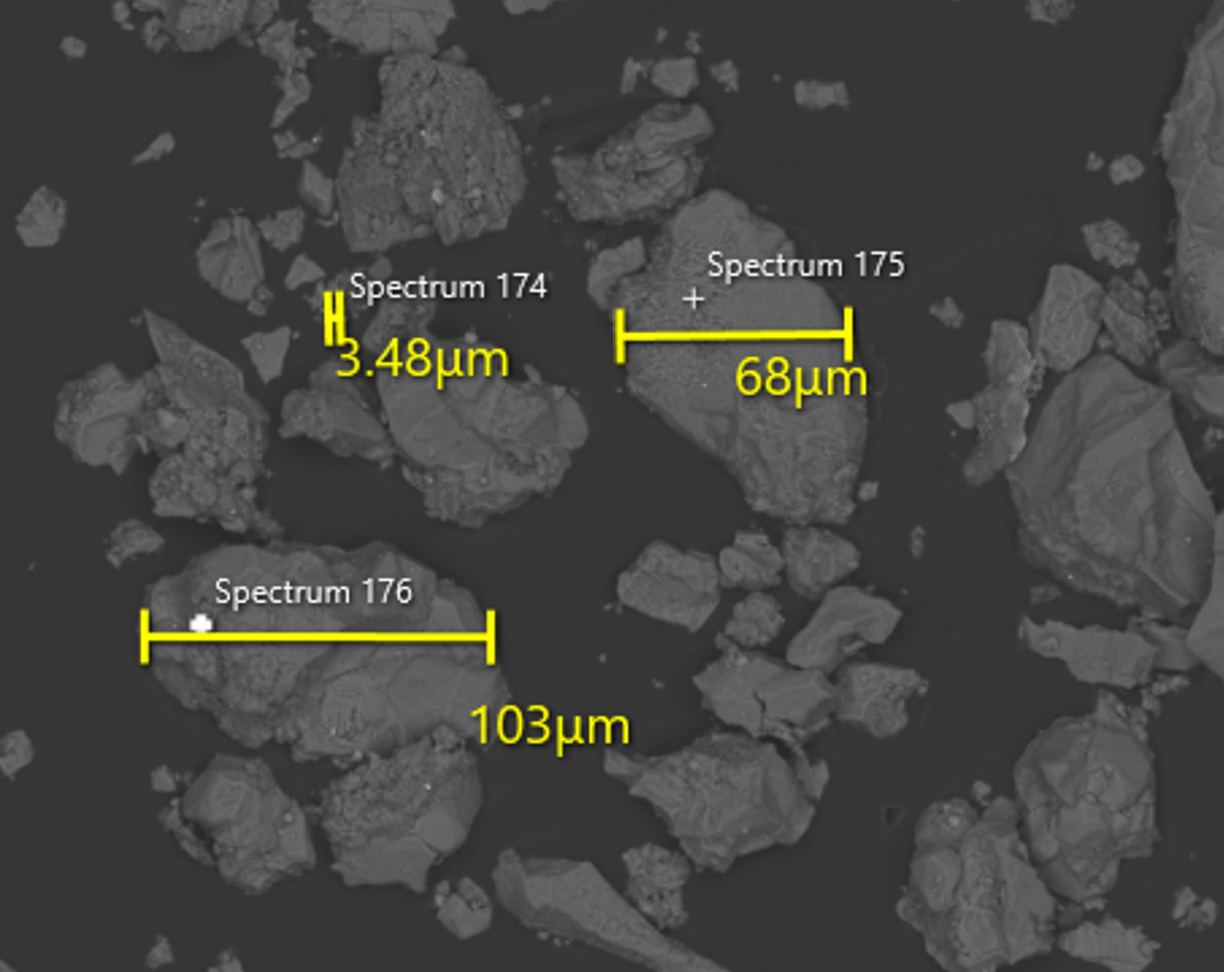 |
|  |  |  |  |
| **DB8** | 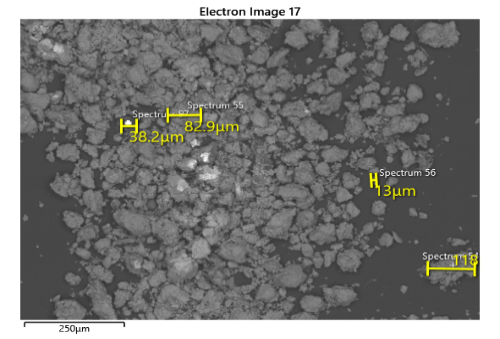 |  | **Fig. S1a.** SEM images of deposition samples from the church of Hagia Sophia (DS1-DS4) and the mosque of Hamza Bey (DB1-DB8) in Thessaloniki, Greece (Sample codes as in Table 1). |

| **DK4** | 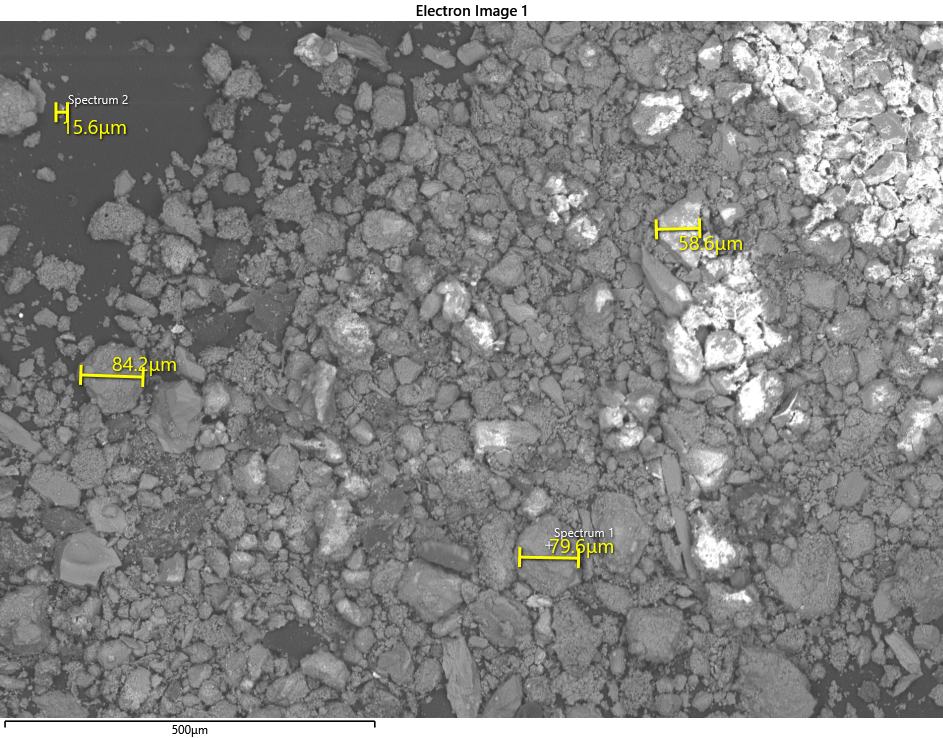 | **DK5** | 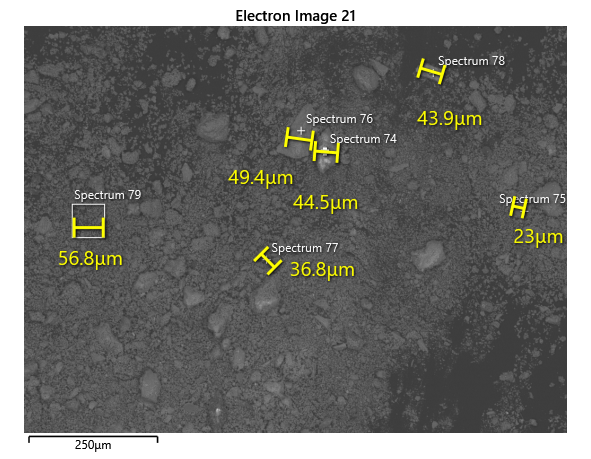 |
| --- | --- | --- | --- |
|  |  |  |  |
| **DK8** | 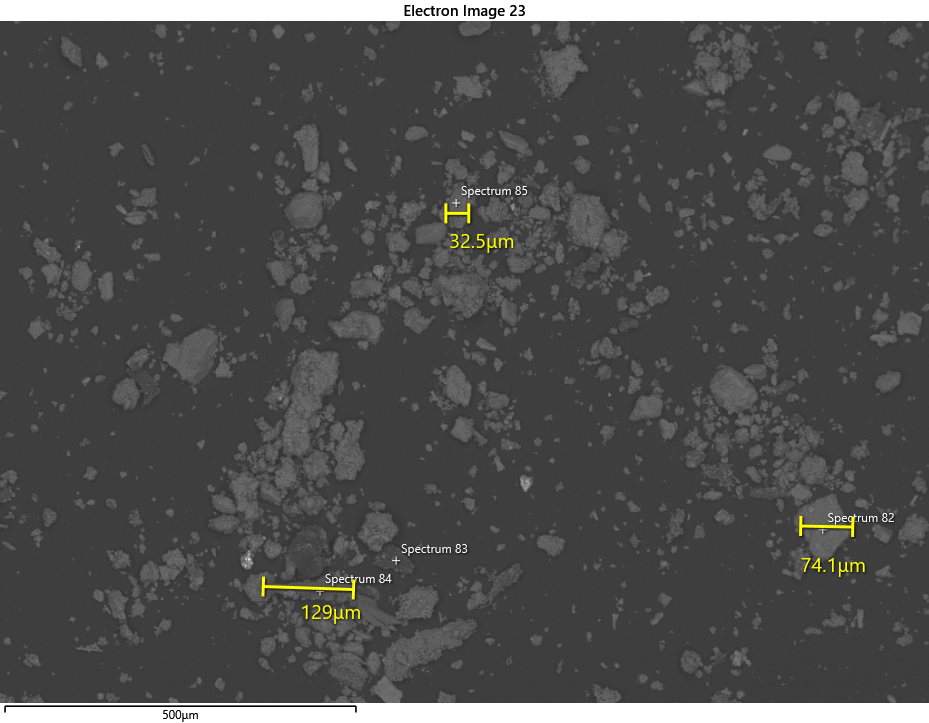 | **DK11** | 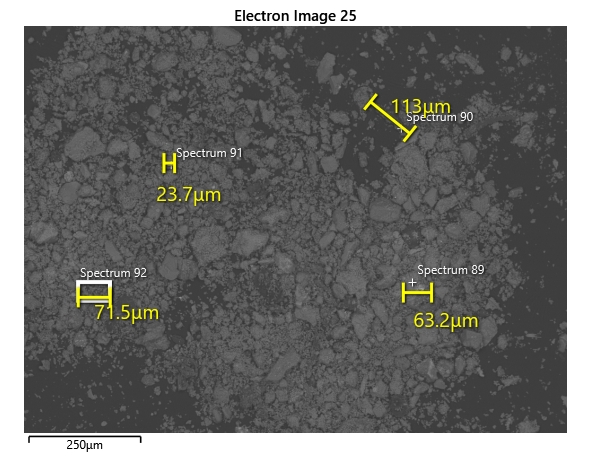 |
|  |  |  |  |
| **DK12** | 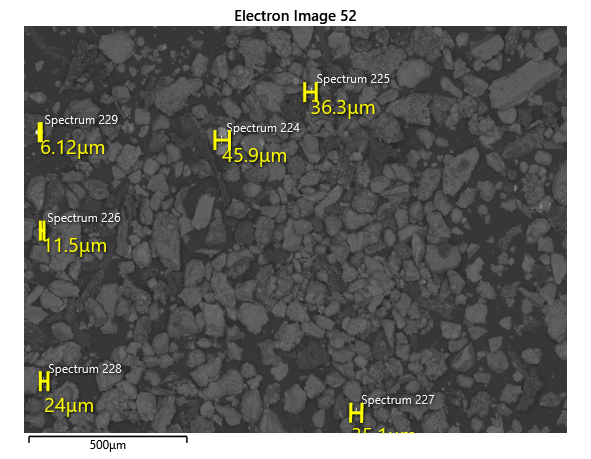 | **DK13** | 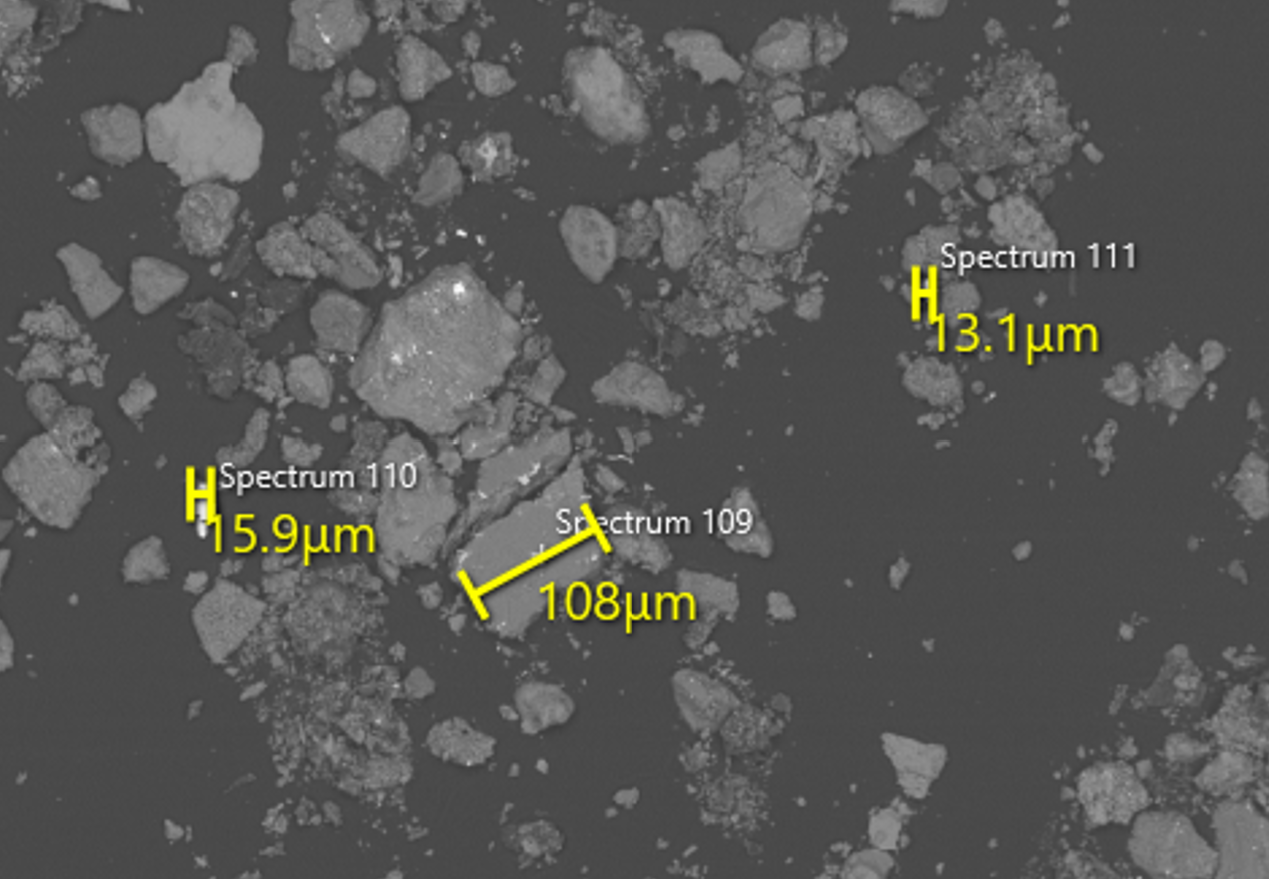 |
|  |  |  |  |
| **DM2a** | **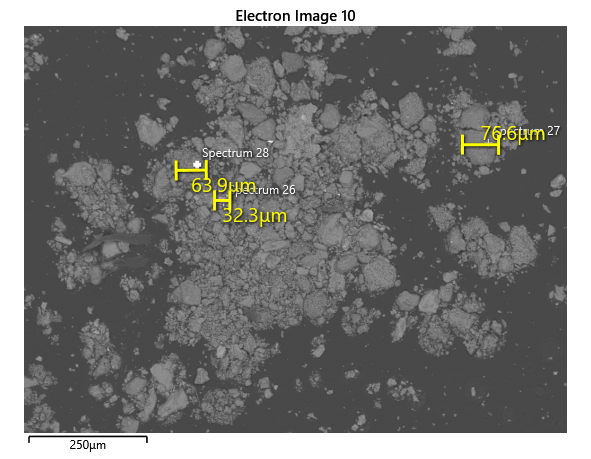** | **DM3a** | 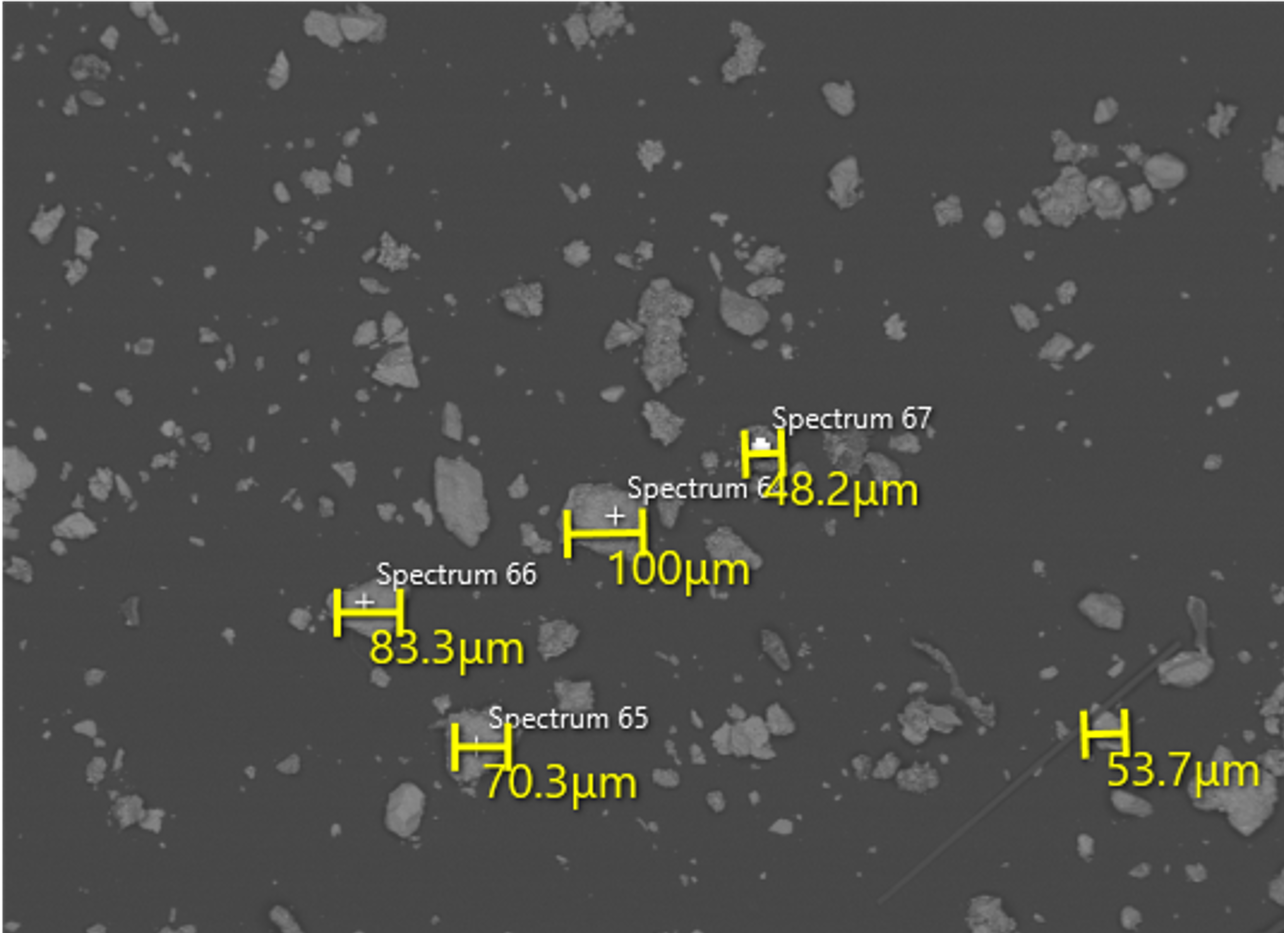 |
|  |  |  |  |
| **DM3b** | 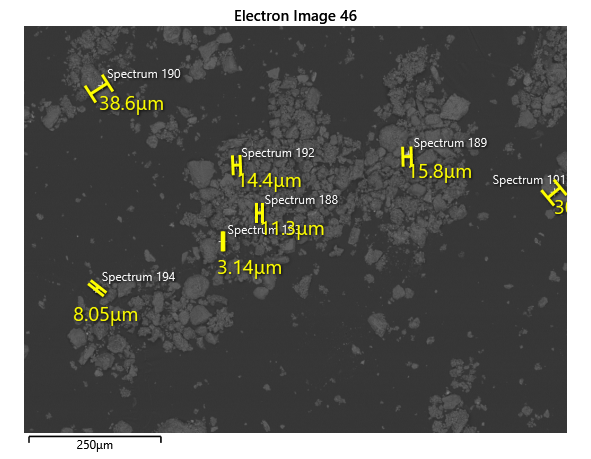 | **DP0** | 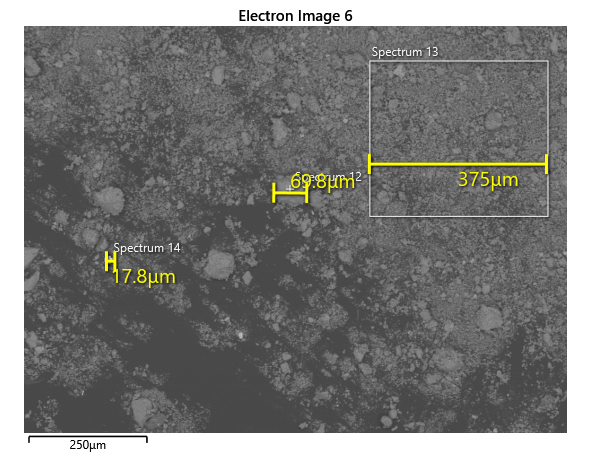 |

**Fig. S1b.** SEM images of deposition samples from the Hadjigeorgakis Kornesios House (DK4-DK13), the church of Archangel Michael Trypiotis (DM2a-DM3b), and the Paphos Gate (DP0) in Nicosia, Cyprus (Sample codes as in Table 1).

|  |  |  |
| --- | --- | --- |
|  |  |  |
|  |  |  |
|  |  |  |
|  |  |  |

**Fig. S2**. Geochemical composition of selected particulate deposition samples obtained by XRF analysis (Sample codes as in Table 1)

**
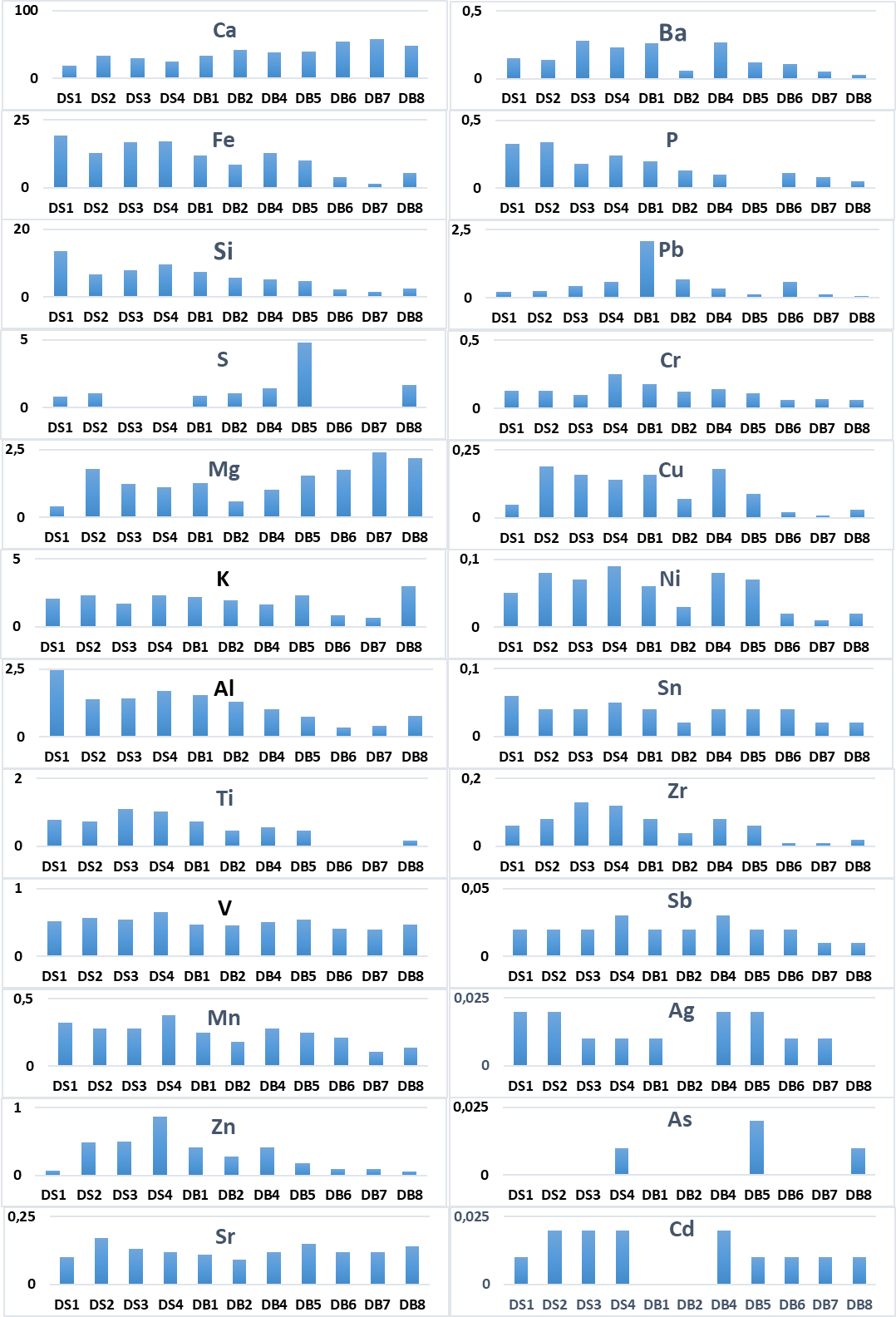
**

**(a)**

**
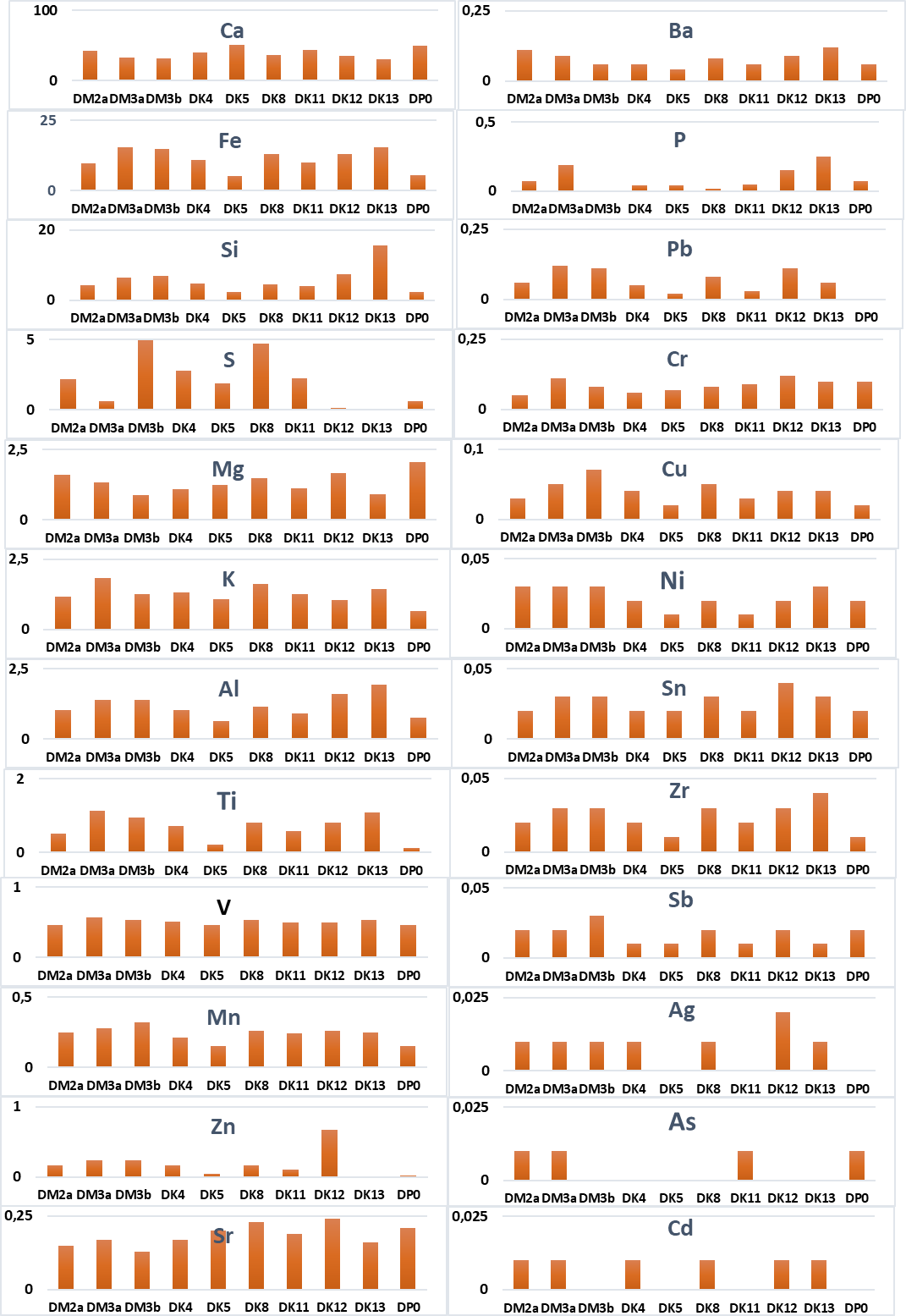
**

**(b)**

**Fig. S3**. Elemental concentrations (% w/w) in deposition samples from Thessaloniki monuments (a) and Nicosia monuments (b) (Sample codes as in Table 1)

**Table S1.** Spearman correlation coefficients between elemental species determined in particulate depositions from monuments of Thessaloniki and Nicosia (N=21).

Only correlation coefficients significant at least at the 95% level are shown. Coefficients significant at the 99% level

are bold-faced.

**Table S2.** Spearman correlation coefficients between ionic species determined in particulate depositions from monuments of Thessaloniki and Nicosia (N=21).

|  | **Cl^-^** | **NO_3_^-^** | **SO_4_^2-^** | **Acetate** | **Formate** | **Oxalate** | **Na^+^** | **K^+^** | **Mg^2+^** | **Ca^2+^** | **NH_4_^+^** |
| --- | --- | --- | --- | --- | --- | --- | --- | --- | --- | --- | --- |
| **Cl^-^** |  |  |  |  |  |  |  |  |  |  |  |
| **NO_3_^-^** | **0.79** |  |  |  |  |  |  |  |  |  |  |
| **SO_4_^2-^** |  |  |  |  |  |  |  |  |  |  |  |
| **Acetate** |  |  |  |  |  |  |  |  |  |  |  |
| **Formate** |  |  |  |  |  |  |  |  |  |  |  |
| **Oxalate** |  |  |  |  |  |  |  |  |  |  |  |
| **Na^+^** | 0.43 | 0.44 |  |  |  |  |  |  |  |  |  |
| **K^+^** |  | **0.73** |  |  |  |  | 0.55 |  |  |  |  |
| **Mg^2+^** | **0.62** | **0.74** |  |  |  |  | 0.57 |  |  |  |  |
| **Ca^2+^** | **0.73** | **0.88** | 0.53 |  |  |  | 0.60 | 0.67 | **0.63** |  |  |
| **NH_4_^+^** |  |  |  |  |  |  |  |  |  |  |  |

Only correlation coefficients significant at least at the 95% level are shown. Coefficients significant at the 99% level

are bold-faced.

**Table S3a**. Abundance (area%) of the pyrolysis products detected in deposition samples from Thessaloniki’s monuments

| **Compound** | **Hagia Sophia Church** | | | | **Hamza Bey Mosque** | | | | | | |
| --- | --- | --- | --- | --- | --- | --- | --- | --- | --- | --- | --- |
|  | **DS1** | **DS2** | **DS3** | **DS4** | **DB1** | **DB2** | **DB4** | **DB5** | **DB6** | **DB7** | **DB8** |
| Carbamic acid monoammonium salt | **32%** |  |  | <10% |  |  |  |  |  |  |  |
| Ethylamine |  | **48%** |  |  | **63%** |  |  |  | **90%** |  | **60%** |
| 1,2-Propanediamine |  |  | **70%** |  |  | **17%** |  |  |  |  |  |
| Styrene |  |  |  | **16%** |  |  |  |  |  |  |  |
| Isopropyl myristate | <10% |  |  |  |  |  |  |  |  | <10% |  |
| Supraene (squalene) | <10%%% |  |  |  |  |  |  |  |  |  |  |
| Toluene | <10% |  | <10% | <10% |  |  |  |  |  |  |  |
| n-Hexadecanoic acid | <10% | <10% |  | <10% | <10% | **14%** |  |  |  | <10% |  |
| 4-Penten-1-ol |  |  |  |  |  | **22%** |  |  |  |  |  |
| Cyclopropyl carbinol |  |  |  |  |  | **12%** | <10% |  |  |  |  |
| Methyl-hydrazine |  |  |  |  |  |  | **54%** |  |  |  |  |
| 3-Methyl butanal |  |  |  |  |  |  | **25%** |  |  |  |  |
| (S)-L-alanine ethylamide |  |  |  | **43%** |  |  |  | **34%** |  | <10% |  |
| 1-Methoxy-2-propanamine |  |  |  |  |  |  |  | **61%** |  |  |  |
| 3-Methyl-heptane |  |  |  |  |  | <10% |  |  |  |  |  |
| Methyl dodecylamine |  |  |  |  |  |  |  |  |  |  |  |
| Benzene |  |  |  |  |  |  | <10% |  | <10%% |  |  |
| n-Hexymethylamine |  |  |  |  |  |  |  |  |  |  | <10%% |
| Pentanal |  |  |  |  |  |  |  |  |  |  |  |
| 5-Methyl-2-heptamine |  |  |  |  |  |  |  |  |  |  | <10%% |
| Cyclobutanol |  | **13%** |  |  |  |  |  |  |  |  |  |
| 2-Amino-heptane |  |  |  |  |  |  |  |  |  |  |  |
| o-Acetylcitric acid triethyl ester |  |  |  |  |  |  |  |  |  |  |  |
| cis-9-Hexadecanal |  |  |  |  |  |  |  |  |  |  |  |
| (S)-(+)-1-cyclohexylethamine |  |  |  |  |  |  |  |  |  |  | <10%% |
| 1,3-Hexadiene |  |  |  |  |  |  | <10% |  |  |  |  |
| Acetic acid |  |  |  |  |  |  |  |  |  |  |  |
| 1-Undecane |  |  |  |  |  |  |  |  |  |  |  |
| Bicyclo[4.2.0]octa-1,3,5-triene |  |  |  |  |  |  | <10% |  |  |  |  |
| Hexanal | <10% |  |  |  |  |  |  |  |  |  |  |

**Table S3b**. Abundance (area%) of the pyrolysis products detected in deposition samples from Nicosia’s monuments

| **Compound** | **Hadjigeorgakis Kornesios House** | | | | | | | **Archangel Michael Trypiotis Church** | | | **Paphos Gate** |
| --- | --- | --- | --- | --- | --- | --- | --- | --- | --- | --- | --- |
|  | **DK4** | **DK5** | **DK8** | **DK11** | **DK12** | **DK13** | **DM2a** | | **DM3a** | **DM3b** | **DP0** |
| Carbamic acid monoammonium salt |  |  | **95%** |  |  |  | **48%** | |  |  | **40%** |
| Ethylamine |  |  |  |  |  |  |  | |  |  |  |
| 1,2-Propanediamine |  |  |  |  |  |  |  | |  |  | <10% |
| Styrene |  |  |  |  |  |  |  | |  |  |  |
| Isopropyl myristate |  | **29%** |  |  |  |  |  | |  |  | <10% |
| Supraene (squalene) |  | <10% |  |  | **19%** |  |  | |  |  | <10% |
| Toluene |  |  |  |  |  | <10% |  | | <10% |  |  |
| n-hexadecanoic acid |  |  |  |  | <10% | <10% |  | |  |  | <10% |
| 4-penten-1-ol |  |  |  |  |  |  |  | |  |  |  |
| Cyclopropyl carbinol |  |  |  |  |  | **24%** |  | |  | <10% |  |
| Methyl-hydrazine |  |  |  |  |  |  |  | |  |  |  |
| 3-methyl-butanal |  |  |  |  |  |  |  | |  |  |  |
| (S)-L-alanine-ethylamide |  | **47%** |  |  | **49%** |  |  | | 10% | **57%** | **12%** |
| 1-Methoxy-2-propanamine |  |  |  |  |  |  |  | |  |  |  |
| 3-Methyl-heptane |  |  |  |  |  |  |  | |  |  |  |
| Methyl dodecylamine |  |  |  | **52%** |  |  |  | |  |  |  |
| Benzene |  |  |  |  |  |  |  | |  |  |  |
| n-Hexymethylamine |  |  |  |  |  |  | 10% | |  |  |  |
| Pentanal |  |  |  |  |  |  |  | | **15%** |  |  |
| 5-methyl-2-heptamine |  |  |  |  |  |  |  | | 10% |  |  |
| Cyclobutanol |  |  | <10% |  |  |  | <10% | | <10% |  |  |
| 2-amino-heptane |  |  |  |  |  |  |  | |  |  | **11%** |
| o-acetylcitric acid triethyl ester |  |  |  |  |  |  |  | |  |  |  |
| cis-9-hexadecanal |  |  |  |  |  |  |  | |  |  | <10% |
| (S)-(+)-1-cyclohexylethamine | **98%** |  |  |  |  |  |  | | <10% | <10% |  |
| 1,3-Hexadiene |  |  |  | <10% |  |  |  | |  |  |  |
| Acetic acid |  |  |  |  |  | **47%** |  | | <10% |  |  |
| 1-Undecane |  |  |  |  |  | <10% |  | |  |  |  |
| Bicyclo[4.2.0]octa-1,3,5-triene |  |  |  | <10% |  |  |  | |  |  |  |
| Hexanal |  |  |  |  |  |  |  | |  |  |  |
